# Predictors of Weight Loss and Weight Loss Maintenance in Children and Adolescents With Obesity After Behavioral Weight Loss Intervention

**DOI:** 10.3389/fpubh.2022.813822

**Published:** 2022-03-25

**Authors:** Alisa Weiland, Lena Kasemann Nannette, Stephan Zipfel, Stefan Ehehalt, Katrin Ziser, Florian Junne, Isabelle Mack

**Affiliations:** ^1^Department of Psychosomatic Medicine and Psychotherapy, University Medical Hospital, Tübingen, Germany; ^2^Public Health Department of Stuttgart, Stuttgart, Germany; ^3^Department of Psychosomatic Medicine and Psychotherapy, University Hospital Magdeburg, Otto von Guericke University Magdeburg, Magdeburg, Germany

**Keywords:** adiposity, obesity, overweight, predictors, children, adolescents, weight loss, weight loss maintenance

## Abstract

**Objective:**

Predictors of body weight loss (BWL) and body weight loss maintenance (BWLM) after behavioral weight loss intervention are well-investigated in adults. Less is known for children and adolescents and a systematic overview on the topic for this age group was aim of the review.

**Methods:**

A systematic research according to PRISMA guidelines using several databases was performed. The outcome was the BMI z-Score of longitudinal studies. The extracted predictors were classified in clusters (Physiology, Behavior, Psychology, Environment) and compared with a theory-driven model based on international guidelines and known predictors for adults.

**Results:**

Out of 2,623 articles 24 met the eligibility criteria, 23 investigating BWL and 8 BWLM. The expected key predictor in research for adults “Behavior” was hardly investigated in children. The most examined cluster was “Physiology” with the most significant predictors, in particular genetics (BWL) and blood parameters (BWLM). Factors in the cluster “Psychology” also predicted BWL and BWLM. The cluster “Environment,” which was highlighted in most intervention guidelines, was neglected in studies regarding BWLM and hardly investigated in studies with BWL.

**Conclusion:**

The comparison with the theory-driven children model outlined research gaps and differences between predictors for adults and children providing further direction of research.

**Systematic Review Registration:**

http://www.crd.york.ac.uk/PROSPERO/, identifier: CRD42020200505.

## Introduction

Obesity and its associated comorbidities are a key public health problem. Most dramatically, childhood overweight and obesity have also become a global epidemic, and many of these children have overweight and obesity in adulthood (1). Overweight implies a major increase in the amount of body fat in comparison with lean body mass. In adults a body mass index (BMI) ≥25 kg/m^2^ is defined as overweight whereas a BMI ≥30 kg/m^2^ is defined as obesity (1).

In children, these cut-offs are not appropriate since their body composition varies between sex and according to their age. As a result, the BMI classifications for children and adolescents are determined relative to peers of the same age and sex using percentiles or the BMI standard deviation score (BMI z-scores). According to international benchmarks, overweight is defined as a BMI ≥85th percentile and <95th percentile, and obesity ≥95th percentile for children and adolescents of the same age and sex (2).

The underlying cause of obesity is an imbalance between energy intake and energy expenditure in favor of the former. The reasons which lead to energy imbalance are multifactorial and include, besides others, genetic and family factors (3). There are different inpatient and outpatient therapies to treat obesity in children and adolescents. These mainly target changes in the fields of behavior, nutrition and physical activity (3). In clinical practice, the success of those interventions is mostly measured by the change of the BMI in adults and the change of the BMI z-score in children (2). However, measurements only relying on body height and weight are unable to deliver information on body composition, the latter being the better indicator for the overall health status (4–6). Thus, body weight changes should be ideally always discussed in relation to shifts in body composition(4–6).

Nevertheless, body weight loss (BWL) and especially body weight loss maintenance (BWLM) remain a challenge. The major reason for this is the difficulty of changing lifestyle behaviors permanently and not just for a short period of time (3). Underlying barriers are a combination of biological (homoeostatic), environmental, and behavioral factors (7). Despite the factors making BWL and BWLM challenging, some children, adolescents and adults are more successful in it than others. This raises questions about possible factors predicting weight loss success, for greater understanding and explanation but also for potential tailoring of treatment options (8–10).

These predictors can be simply subdivided into four clusters of investigations depending on the main focus of the researchers and research groups (11):

(a) Physiology: demographics, diseases, blood parameters, genetics and taste.
(b) Behavior: eating behavior and physical behavior which combined balance energy intake and expenditure.
(c) Psychology: motivation, compliance and cognitive resources.
(d) Environment: social environment and treatment conditions.

**Figure 1:**
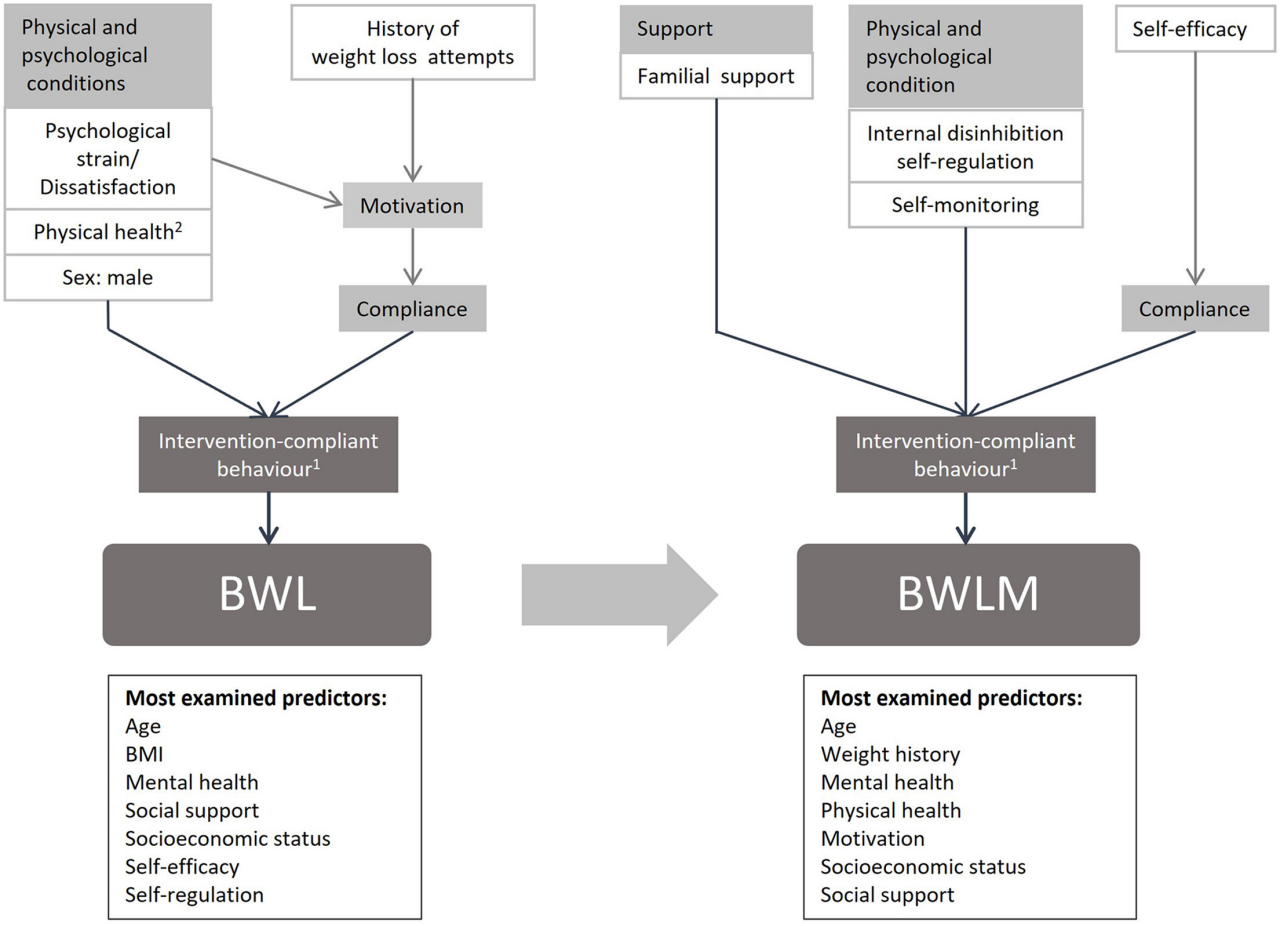
Model of predictors for BWL and BWLM in adults. 1: Eating behavior (low energy intake), Physical activity (high energy expenditure), Sedentary behavior (low energy expenditure); these factors are all influenced by pre-existing behavior (e.g. dietary restrain). 2: Blood pressure, Typ 2 Diabetes, Sleep apnoea, Somatization, Hypercholesterinaemia, Cardiac metabolic complications.

For the adult sector many longitudinal predictors have been already reported in a high quality systematic review (11). Those are summarized in the theory-driven adult model shown in Figure 1. Both, BWL and BWLM, are only successful, if compliance to the behavior change intervention is maintained. Key components of behavioral predictors are lifestyle factors including a healthy diet and eating behavior, moderate physical activity levels and decreased levels of sedentary behaviors (11, 12). For BWL, an imbalance between energy intake and energy expenditure in favor of the latter is necessary. In contrast, for BWLM a balance between energy intake and energy expenditure is required. Factors that influence the possibility of an intervention-compliant behavior differ between BWL and BWLM. Interestingly, environmental predictors like support are only important in BWLM, whereas several physiological conditions such as pre-existing diseases or the sex matter only for BWL (12). Also, psychological conditions like psychological strain and health are predictive for a successful BWL, whereas self-monitoring and self-regulation abilities are important for BWLM (11). In addition, different factors that affect compliance itself were found for BWL and BWLM. In adults, dissatisfaction and history of weight loss attempts are important for BWL, whereas self-efficacy is an important psychological predictors for BWLM (12). As yet, it is unclear whether or not these predictors are similar or differ between children and adults and a thorough systematic review on this topic is missing in the literature.

**Figure 2:**
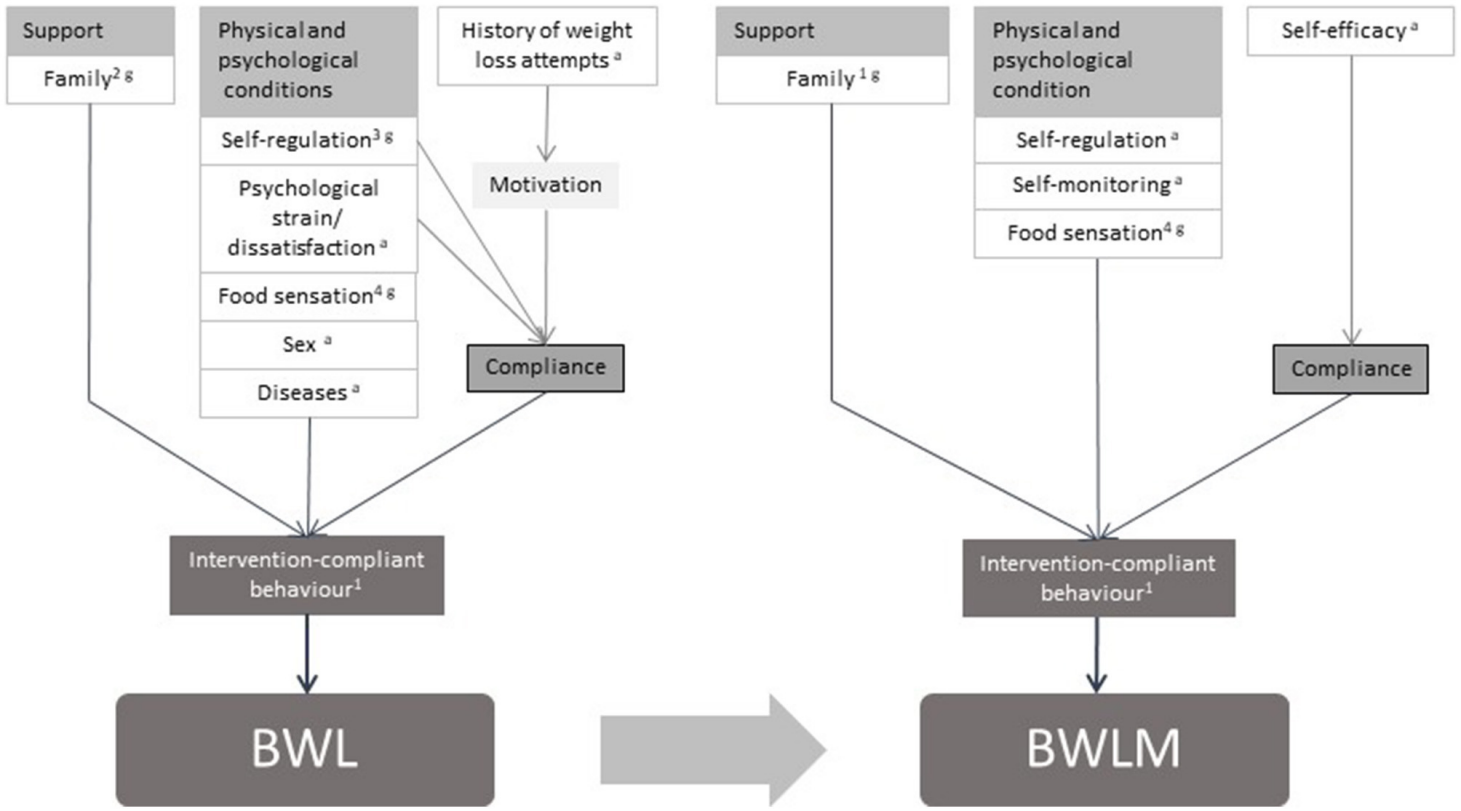
Model of predictors for BWL and BWLM in children. Theory-driven model of predictors for body weight loss and body weight loss maintenance in children and adolescents (11–13). 1: Eating behavior (low energy intake), Physical activity (high energy expenditure), Sedentary behavior (low energy expenditure); these factors are all influenced by pre-existing behavior. 2: The family should be included in the treatment, their behavior should not be different than the child with excess weight, they should offer healthy food, eat as a family. 3: Stimulus control. 4: Recognition and respect of food sensation. a: Adult's model. g: Review of guidelines for treatment of pediatric overweight and obesity (included 17 guidelines, all recommendations mentioned in at least 30% of these guidelines have been taken into account), only additional added factors were marked with a “g”.

Thus, based on the model for adults (11) and complemented by 17 international high-quality guidelines for the treatment of childhood obesity (13), a theory-driven model for children was created (Figure 2). In the model factors were reported when at least present in 30% of the 17 guidelines summarized in the systematic review by Pfeiffle et al. (13). For better comparison between the results of children and adults the same clusters were chosen as in Varkevisser et al. (11).

Similar to the adult model (Figure 1), the theory-driven children model (Figure 2) contains predictors from all four clusters. The aim of this study was to compare the theory driven predictors for successful children's BWL and BWLM based on the existing findings in adults and the international guidelines with the predictors extracted by this systematic review. The hypotheses are summarized below:

- Hypothesis i: The key-predictor for children's BWL and BWLM is the intervention-compliant behavior represented by eating behavior, physical activity and sedentary behavior.
- Hypothesis ii: Support by the social environment, especially parent's support, is among the most important predictors for children's BWL and BWLM.
- Hypothesis iii: Physical and psychological conditions such as food sensation, executive functions, strain and dissatisfaction, or sex and diseases predict BWL, whereas for BWLM only food sensation and the executive functions self-monitoring and self-regulation are positive predictive.
- Hypothesis iv: Compliance predicts successful BWL and is influenced by previous experiences, motivation and executive functions. Compliance also predicts BWLM with self-efficacy being the most important influencing factor.

## Methods

### Literature Information Sources and Search Strategy

This review was developed and executed according to the Preferred Reporting Items for Systematic Reviews and Meta-Analyses (PRISMA) guidelines (14) to identify all relevant studies examining predictors of BWL and BWLM in children and adolescents with obesity after behavioral weight loss intervention. The databases PubMed, Cochrane Library and Web of Science (core collection) were applied. The research was carried out on PubMed on 27/04/2020 and on Cochrane Library and Web of Science on 06/05/2020. The protocol of this systematic review is registered at the PROSPERO platform with the registration number CRD 4202 0200 505.

The search strategy consisted of four elements:

1. Obesity.
2. Behavioral Weight Loss Intervention.
3. Predictors for BWL or/and BWLM.
4. Definition of age-Group for the Intervention's Participants (Children, Adolescents).

The full search strategy is documented in the Supporting Text S1.

### Eligibility Criteria

To define the eligibility criteria the five PICOS dimensions were used i.e., Participants (P), Interventions (I), Comparators (C), Outcomes (O), Study design (S) (15).

*Participants:* Children and Adolescents with obesity defined as body mass index (BMI) ≥95th percentile (2) of both sexes and all ethnicities on average aged 0–18 years (16). Studies that examined only patients with a specific disease other than obesity (e.g. weight reduction in patients with asthma) were excluded.

*Interventions:* Behavioral, moderate BWL intervention programs consisting of multidisciplinary approach (diet, physical activity, behavior) in inpatient or outpatient settings, lasting 1 to 12 months and with a BMI z-score reduction of at least 0.2 at the end of the study.

*Comparators:* Studies with and without control groups were included.

*Outcomes:* The outcome was the change of the BMI z-score. As data outcome regressions and correlation analysis were included, the latter only in case of a non-predictive result. The minimum requested follow-up length of studies examining BWLM was 3 months (17, 18).

*Study design:* Randomized controlled trials, randomized non-controlled trials (RTs) and uncontrolled before-after intervention without group comparison (BA).

Peer-reviewed original articles written in English or German were included.

### Study Selection, Data Collection and Organization

The articles were screened independently by two of the authors (AW, LK) in three steps. First, duplicates and reviews were removed, secondly, title and abstracts were screened and lastly, full-text screening was carried out. The overall interrater-reliability was 0.98. After every step, conflicts were discussed and if necessary a third person was consulted (I.M.; 2.0% of the entire search results were evaluated in discussion). The selected studies were categorized in two groups: Predictors of BWL (Group 1) and Predictors of BWLM (Group 2).

### Data Items and Statistics

The following information was extracted from each included article: year of publication, country of origin, study type, sample size, interventions characteristics, setting (in/outpatient), intervention characteristics (diet, sports, behavioral therapy), intervention/follow-up length, parent's involvement, sample characterization including age, gender, z-score BMI outcome measures, investigated predictors, statistical method and kind of prediction. Corresponding authors were contacted for missing or unclear information. Twenty One percent of the authors responded.

### Risk of Bias

To verify the quality of the included studies the modified quality assessment tool (11) was utilized, originally used by (19). The assessment tool addresses six rating criteria. Each fulfilled criterion was rewarded with one point, allowing a maximum of six points in total. The six elements are defined as follows:

(i) Representativeness of the cohort at baseline: At least 80% of the cohort participated at baseline or a not selective non-response; (ii) representativeness of the cohort at follow-up: Response rate at follow-up at least 80% of the cohort or a not selective non-response; (iii) valid and reliable measurements of predictors: Test-retest correlation ≥0.80 or κ/ICC ≥0.70 for each measurement (fulfilled: points per measured predictors = 1/number of measured predictors, not fulfilled/not reported: 0 points; e.g., five predictors were measured, then 0.2 points for each reliable measured variable could be given); (iv) valid and reliable measurements of weight: Measurement with an objective tool or questionnaire; Test-retest correlation ≥0.80 or κ/ICC ≥0.70; (v) proper sample size: Sample size ≥(10 multiplied with number of independent variables); (vi) proper statistical methods: Appropriate statistical methods, if necessary adapted in case of disturbing.

## Results

### Study Selection and Categorization

**Figure 3:**
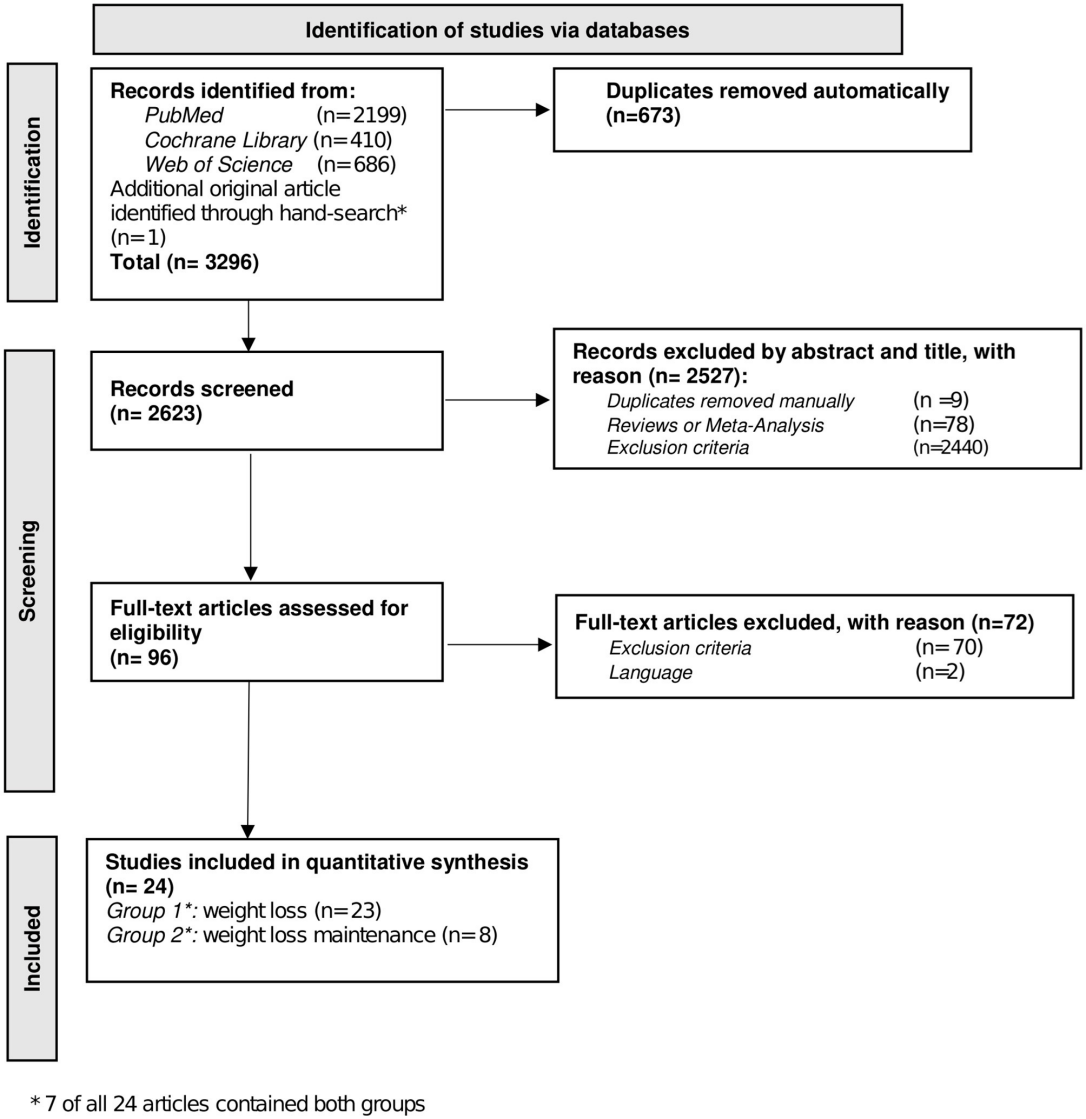
Screening-process. Flow chart of Preferred Reporting Items for Systematic Reviews and Meta-Analyses (PRISMA) systematic search.

Figure 3 shows the literature search process. Out of 2,623 articles remaining after duplicate removal, 24 studies were identified for inclusion. Out of these, 23 papers were categorized into group one: BWL (20–42). One article only focused on BWLM (43). This article, together with seven other papers which also investigated BWL (21, 23–25, 30, 40, 41) were eligible for group 2: BWLM.

### Risk of Bias

The summary of the risk of bias of the studies is presented in Table 1. All studies except one got no point for the criterium representativeness of the exposed cohort due to not reporting or not-fitting of the criterium (21 studies) and participation rate under 80% (two studies). Drop-out rate and validity and reliability of measurements of predictors scored moderately well with 50.00% and 66.17% of the studies, that met the criterium. In validity and reliability of weight measurement, appropriate sample size and appropriate statistic models the studies performed very well-achieving 87.50, 87.50 and 83.33%. Supplementary Material 1 shows the quality rating for each study. Supplementary Material 2 shows the risk of bias in detail.

**Table 1:** Risk of bias.

**Criteria**	**Percentage of articles that meet the criterion**
Representativeness of the exposed cohort	4.17
Drop-out rate	50.00
Validity and reliability of measurements of determinants	66.17
Validity and reliability of weight measurement	87.5
Appropriate sample size	87.5
Appropriate statistic models	83.33
Average amount of fulfilled criteria in total	63.11

*Risk of bias of all studies rated with the Quality Assessment Tool (43)*.

### Summary of Study Characteristics

#### Study Characteristics Group 1: BWL

An overview of the characteristics across studies is provided in Table 2. The most popular study type was a before-and-after comparison without a control group (*n* = 17). Seven articles were non-randomized controlled trials. The papers were conducted mainly in European countries: Belgium (*n* = 2), Denmark (*n* = 1), Italy (*n* = 1), Germany (*n* = 9), Switzerland (*n* = 1), Spain (*n* = 4), Netherlands (*n* = 1) or Czech Republic (*n* = 1), but also in the United States of America (*n* = 3). The median age was 12.5 years. Nineteen studies included both sexes, with 43.5% (median) boys. The median z-BMI at baseline was 2.5 and ranged from 2.0 to 4.8 (z-score 1.645 ? 95th percentile; BMI ≥95th is defined as obesity). In four studies the parents were involved in the intervention (21, 23, 25, 40).

**Table 2:** Characteristics across studies for all studies, subgroup BWL and subgroup BWLM.

**Sample size**	**Median**	**IQR**	**Minimum**	**Maximum**
**All studies (*****n*** **=** **24)**				
Sample size (r = 24)	150.0	[101.8–200.0]	12.0	12,305.0
Sex (% boys) (r = 23)	43.2	[38.9–48.2]	13.3	53.3
Age (years) (r = 20)	12.5	[11–14.2]	9.2	14.9
z-BMI (r = 24)	2.5	[2.4–3.1]	1.9	4.8
**BWL (*****n*** **=** **23)**				
Sample size (r = 23)	150.0	[92.5–201.0]	12.0	12,305.0
Sex (% boys) (r = 22)	43.5	[38.5–48.2]	0.0	100.0
Age (years) (r = 20)	12.5	[11.0–14.2]	9.2	14.9
z-BMI (r = 23)	2.5	[2.4–3.2]	2.0	4.8
**BWLM (*****n*** **=** **8)**				
Sample size (r = 8)	172.0	[150.0–394.0]	74.0	12,305.0
Sex (% boys) (r = 8)	41.8	[33.1–47.9]	13.3	56.2
Age (years) (r = 7)	11.7	[10.5–14.2]	10.4	14.8
z-BMI (r = 8)	2.3	[2.0–2.6]	1.9	4.8

*IQR, interquartile range; n, total number of studies in this group; r, amount of studies reporting the variable (BWLM parameters are baseline values. Thus, they are not necessarily representative for the follow-up population)*.

#### Study Characteristics Group 2: BWLM

Detailed information on study characteristics are provided in the supplements (Supplementary Material 1). Eight studies were included in this group. Except for one study (43), the others also reported BWL along with BWLM. The study types where either non-randomized controlled trials (*n* = 1) or before-and-after comparisons without a control group (*n* = 7). They were conducted in the United States of America (*n* = 2), Italy (*n* = 1), Spain (*n* = 1), Switzerland (*n* = 1) and Germany (*n* = 3). The median age at baseline was 11.7 years, their median z-BMI 2.3 (range: 1.9–4.8) and the proportion of boys was 41.8% (median). The cohort of patients lost to follow up were not characterized (including sex distribution), only sample size in three studies was given with a median of participants (30, 40, 41). Three studies included the parents in the intervention (21, 23, 25). All characteristics across studies are shown in Table 2.

### Summary of Study Outcomes for BWL

In total, 146 different predictors were investigated in the 23 studies. The different predictors were grouped into 16 groups and classified into the four main clusters: Physiology, Behavior, Psychology and Environment. A detailed description of the categorization of the predictors is shown in Supplementary Material 3. Most articles examined Physiological factors (*n* = 17), comprising 75 factors.

In the cluster Physiology the most often examined type of predictor was in the field of genetics (24, 28, 29, 34, 36, 37) and demographics (22, 27, 33, 35, 37, 39, 40).

The second largest cluster was Psychology (*n* = 8) (20, 22, 23, 25, 27, 31, 32, 40). In addition to mental health, areas of motivation, body image and impulse control were investigated. This was the most investigated cluster with 20 investigations (20, 23, 25, 31, 40).

Only a few articles considered the cluster Behavior (*n* = 3) (20–22) and Environment (*n* = 5) (22, 23, 33, 35, 40).

**Figure 4:**
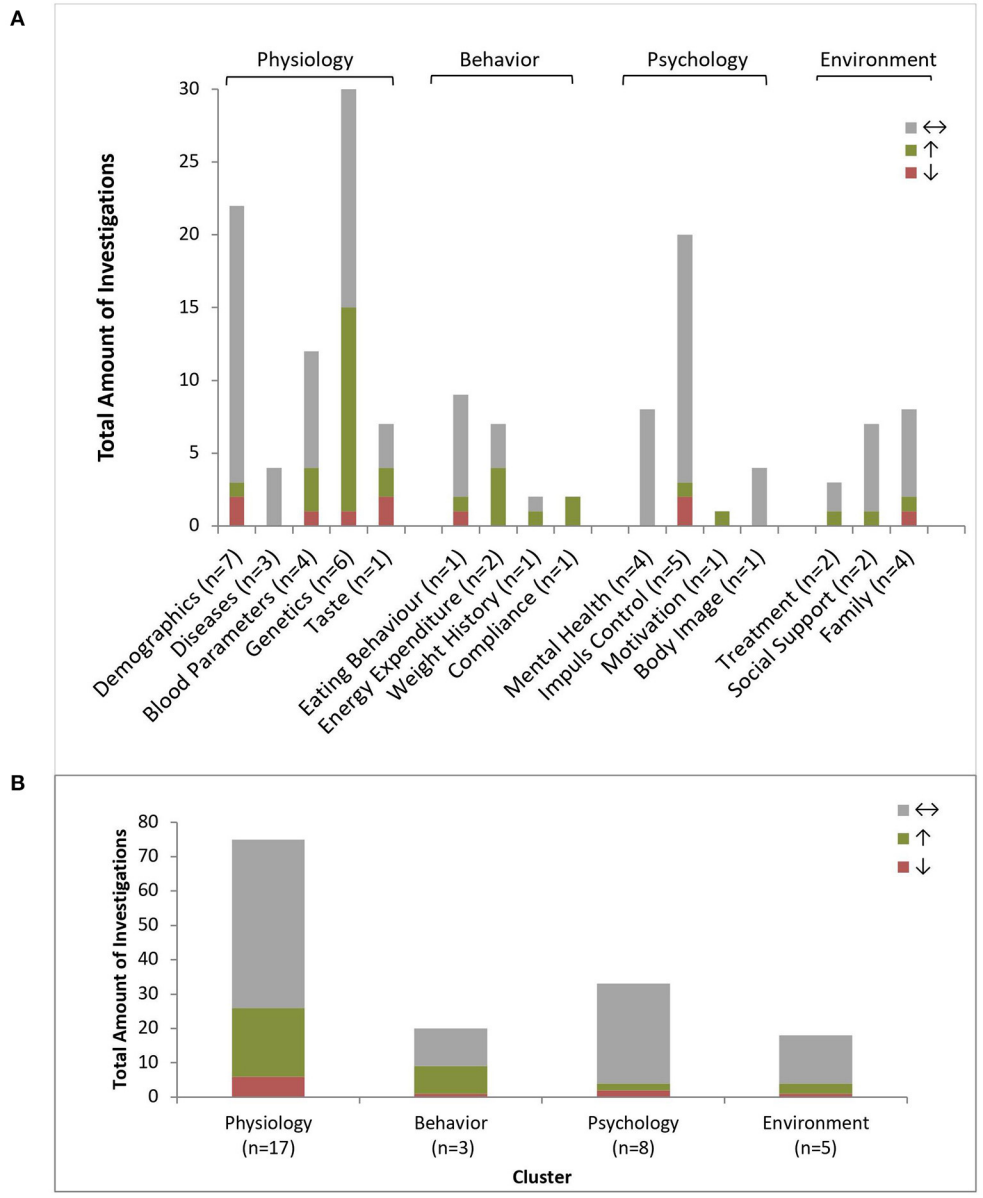
Predictors of body weight loss. Amount of positive (↑), negative (↓) or non-predictive (↔) investigations regarding BWL (*n* = 158) per predictor type **(A)** and grouped by clusters **(B)**; n = number of investigating studies per predictor type (n total = 23).

Figure 4 and Supplementary Material 3 provide an overview about the investigated predictors of the included articles. In short summary there were positive and negative predictors for BWL in every cluster.

#### Physiology – BWL

The cluster Physiology consisted of demographics, diseases, blood parameters, genetics and food sensation (taste). Over all 65% (*n* = 49) of the investigations were non-predictive, 27% (*n* = 20) positive predictive and 8% (*n* = 6) negative predictive. An overview detailing all predictors is given in Supplementary Material 3, the overview over the reports statistics of the predictors is provided in Supplementary Material 1.

Regarding demographics, the data was consistent, finding that neither sex, age (22, 33, 35, 37, 39) nor body size or the initial BMI z-score (22, 35) were predictive for BWL (22, 27, 39). Additionally, birthweight was a non-predictive factor (investigated once). However, delayed puberty resulted in a worse response to BWL intervention. Unclear results were found regarding the socioeconomic status (35, 40).

Diseases including gestational diabetes, asthma and adenovirus 36 did not predict BWL (22, 39, 42).

Most parameters in the blood parameters group were non-predictive. Only baseline leptin levels predicted BWL negatively (30), whereas TSH concentrations and fT3 (41) concentrations predicted BWL positively. Higher C-peptide levels in girls lead to greater BWL (26).

Many investigations analyzed genetic factors. Most investigated genes were non-predictive. A few specific genes were positive predictive (TMEM18, LEPR, APOA1, CETP, TFPI, LTBP1, MMRN1 and PKHD1L1) (28, 29, 34), one negative predictive (MC3R) (37) and for FTO prediction was not consistent (one time positive (29), one time not predictive (28)). For boys the telomere lengths at baseline was also a positive predictor (24).

One study (38) found the ability to taste sweet to be positive predictive for BWL (high sensitivity for sweet leaded to a poorer BWL) whereas the opposite was shown for bitter taste (high sensitivity for bitter leaded to a greater outcome).

#### Behavior – BWL

The cluster Behavior included the groups eating behavior, energy expenditure, weight history, and compliance. Seventy Five percent (*n* = 24) of all behavioral investigations were non-predictive, 22% (*n* = 7) positive predictive and 3% (*n* = 1) negative predictive. Regarding eating behavior, only the daily water intake was positively and soda intake negatively predictive for BWL (22). Two studies investigated energy expenditure as potential predictors. Ball skills and the preexisting physical activity level predicted a successful BWL (20, 22). Manual dexterity and balance skills (20) as well as general eating behavior were non-predictive (21, 22, 40). The findings for body weight history were inconclusive, with recent weight change was reported as positive predictive, the recent weight gain was non-predictive (22). Compliance and adherence to treatment were repeatedly confirmed as a positive predictor for BWL (22).

#### Psychology – BWL

Psychology was divided into four subgroups: mental health, impulse control, motivation and body image. Ninety One percent (*n* = 30) of all behavioral investigations were non-predictive, 3% (*n* = 1) positive predictive and 6% (*n* = 2) negative predictive. There was strong evidence that mental health is non-predictive (22, 27, 32, 40) as well as motivation (22) and body image (27). The research on impulse control showed ambivalent findings. Many studies detected different factors as non-predictive for BWL, more precisely, digit span (23), self-regulation (23, 25), updating abilities and inhibition control (20) and planning and decision making (20, 23). In contrast, Pauli-Pott and colleges reported 2010 that high impulsivity had a negative influence on BWL. Unclear findings were reported regarding attention shifting (20) and inattention (31, 40).

#### Environment – BWL

The cluster Environment was composed of the topics treatment, social support and family. 77.8% (*n* = 14) of all environmental investigations were non-predictive, 16.7% (*n* = 3) positive predictive and 5.6% (*n* = 1) negative predictive.

Mixed findings were presented for treatment condition (22, 35). Apart from the positive predictive factor family encouragement, each examined variable within social support did not predict BWL (22, 33). The familial circumstances were also mostly non-predictive (parent's executive functions, depression and weight) (22, 23, 33, 40). However, having siblings with obesity predicted less BWL (33) and being an only child lead to greater BWL (22).

### Summary of Study Outcomes for BWLM

Eight studies reported 45 investigations for BWLM, grouped into 10 types of predictors which were further grouped in the four familiar clusters: Physiology, Behavior, Psychology, and Environment. A detailed description of the predictors is shown in Supplementary Material 4.

**Figure 5:**
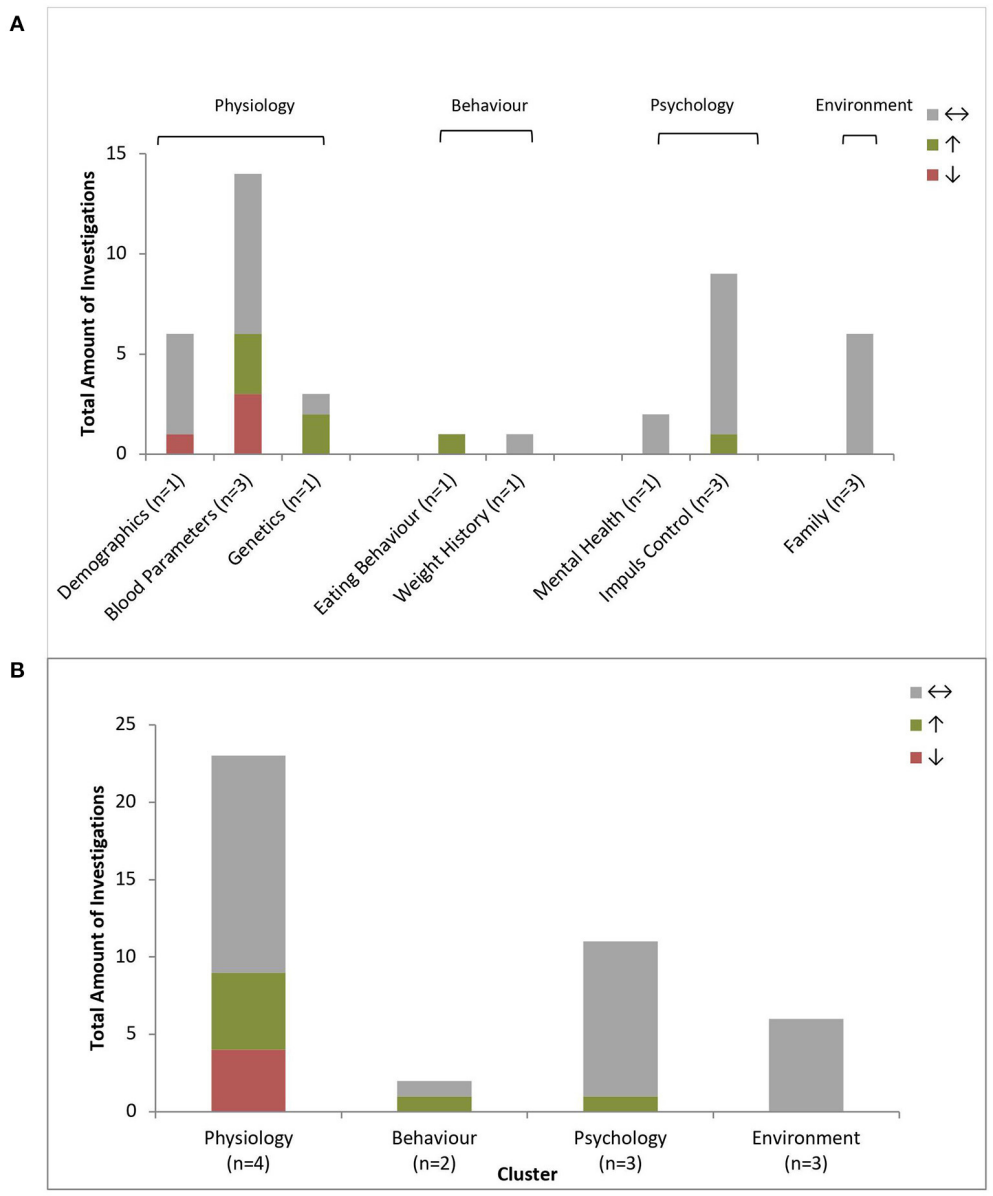
Predictors of Body Weight Loss Maintenance. Amount of positive (↑), negative (↓) or non-predictive (↔) investigations regarding BWLM (*n* = 45) per predictor type **(A)** and grouped by clusters **(B)**; n = number of investigating studies per predictor type (n total = 8).

Half of the included articles dealt with Physiological factors (*n* = 4), comprising of 26 investigations in total regarding physiological factors. Here, the most often examined type of predictors were blood parameters (30, 41, 43) and demographics (43). The second largest cluster was Psychology (*n* = 3) (23, 25, 40). Three investigations considered mental health (40) and eight investigations impulse control (23, 25, 40). Only a few articles considered Behavioral investigations (*n* = 2) (21, 43) and Environmental predictors (23, 40, 43), with six investigating familial circumstances. A detailed overview about the investigations is provided in Figure 5.

#### Physiology – BWLM

Physiological factors included demographics, blood parameters and genetics. BWLM was assessed at different timepoints. A detailed overview over the predictors is provided in Supplementary Material 4.

Sixty-five percent (*n* = 17) of all environmental investigations were non-predictive, 19% (*n* = 5) positive predictive and 15% (*n* = 4) negative predictive. Consequently, predictors grouped as demographics were non-predictive for BWLM (43). Only delayed puberty predicted BWLM negatively (43).

Mixed results were found for blood parameters. Neither the insulin level (43), blood lipids (39) and blood pressure (43) at baseline, nor the decrease in fT4 during intervention (41) were significant predictors for BWLM. Negative predictors were a TSH and fT3 decrease during Intervention (41). Insufficient evidence was found to draw a conclusion about leptin levels (30, 43). Only in boys, BWLM was positively predicted by their telomere lengths at baseline (24).

#### Behavior – BWLM

Two studies investigated environmental factors, with one study reporting non-predictive and one positive predictor. Behavior is grouped in eating behavior and weight history. One study found that BWLM was predicted by the appetitive trajectories group: A high satiety group was better in maintaining their weight than the high emotional eating or the high food responsive groups (21). Weight change during the intervention had no predictive influence (43).

#### Psychology – BWLM

Ninety one percent (*n* = 11) of all environmental investigations were non-predictive, 9% (*n* = 1) positive predictive and none negative predictive. The psychological cluster of BWLM consisted of mental health and impulse control. None of the predictors grouped in mental health were predictive for BWLM (40). Most of the investigated variables regarding impulse control did not predict BWLM (23, 25, 40). However, planning and decision making measured through the Wisconsin Card Sorting Test predicted long-term BWLM positively, but not in mid-term BWLM (23).

#### Environment – BWLM

One hundred percent (*n* = 6) of all environmental investigations were non-predictive. The environmental group only contained family characteristics. None of them significantly predicted BWLM (23, 40, 43).

## Discussion

This review thoroughly summarizes studies analyzing predictors for BWL and BWLM in children and adolescents after a behavioral BWL-intervention. The predictors are grouped into the clusters Physiology, Behavior, Psychology and Environment. The heterogeneity of investigated predictors along with their outcomes was very high. Most predictors were examined for BWL but not BWLM.

**Figure 6:**
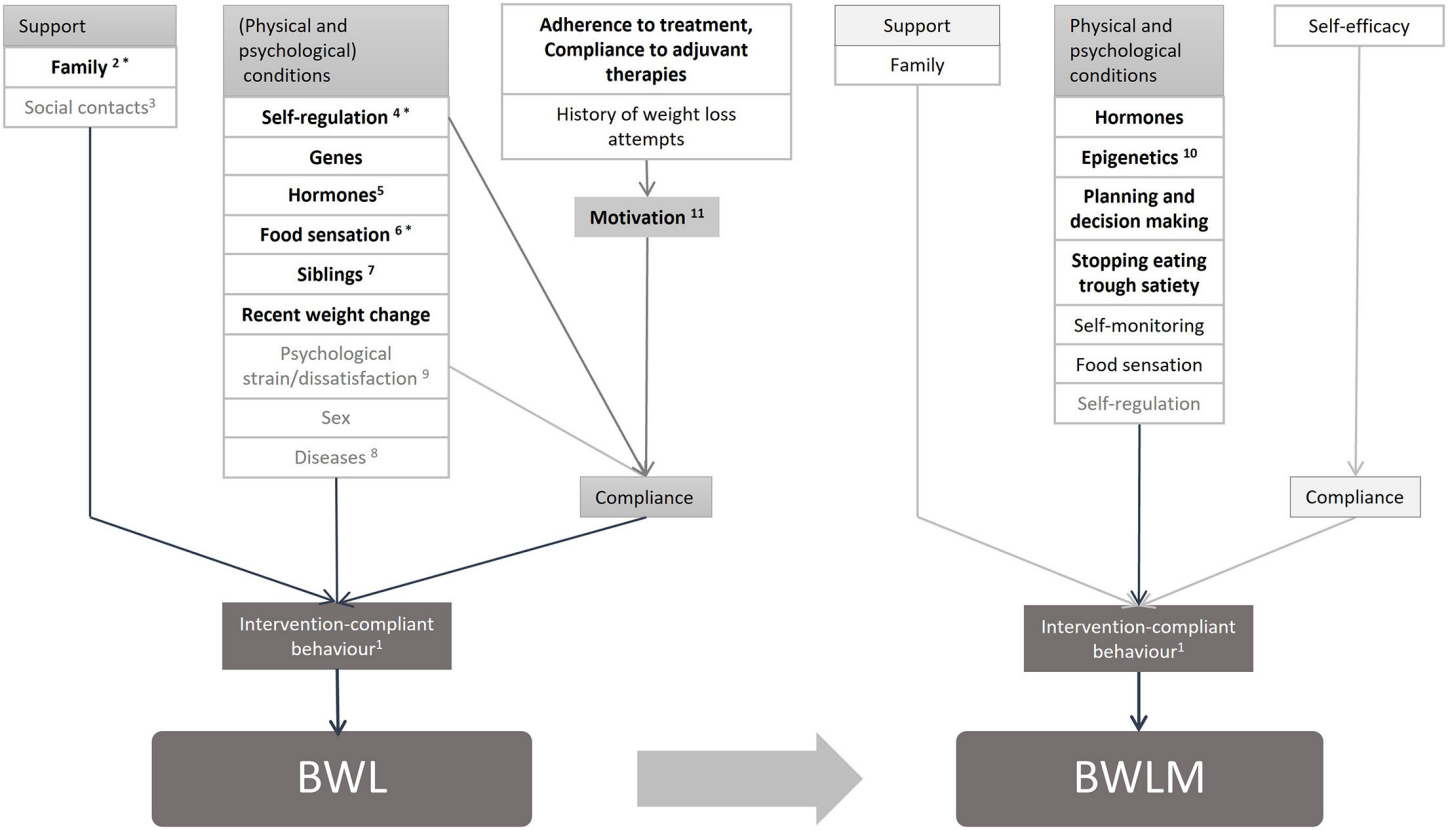
Children's model for implemented predicting factors on BWL and BWLM. 1: Physical activity (high energy expenditure)* Preexisting physical activity/ competences (BWL, BWLM), Eating behavior (low energy intake) (BWL, BWLM), Sedentary behavior (low energy expenditure), Liquid intake (BWL, BWLM), Conduct problems; these factors are all influenced by preexisting behavior; 2: Family encouragement to the project; 3: Examined factor: Social integration; 4: Examined factors: Inattention, Impulsivity; 5: including also Delayed puberty; 6: Taste; 7: Only child, Obese siblings; 8: examined diseases: Asthma, Adenovirus 36 Antibodies, Mental health [Conduct problems, Eating disorder, Mental health, Stress (sleep quality)] (BWL, BWLM); 9: examined factors: Concerns about body weight shape; Stress (quality of sleep); 10: Telomere length boys, Telomere length girls; 11: Child's Motivation. (Bold and black printed: predictive, black printed: not examined or not reported, gray printed: examined, but non-predictive; *predictive and listed in the children's model).

The research results were added into and compared with the theory driven predictor-model for children for graphical visualization and for outlining research gaps (Figure 6).

The first assumption, that intervention compliant behavior is a key predictor for BWL and BWLM, could not be verified. Underlying influencing factors including preexisting eating behavior, physical activity and sedentary behavior were mainly non-predictive (20–22). The only possible components to maintain body weight seem to be preexisting physical activity and motor competences (20, 22). Apparently, even though the change of these behaviors is very important to lose body weight (44) the preexisting behavior did not have great impact on the short-term success of the therapy. The question remains, wether preexisting behavior effects BWLM.

Social support, especially parental, is mentioned as a key-factor for BWL in several guidelines for obesity treatment (13). Thus, the second hypothesis stated that social support is a predictor for BWL. However, this could not be sufficiently proven. Only two studies investigated some social support parameters for BWL, in which one study identified parents support as positive predictive for BWL, whereas the other found that social integration did not influence BWL. For BWLM it was not investigated at all. Nonetheless, systematic reviews and meta-analysis were able to show positive effects of parent involvement in weight loss interventions or weight related health interventions for children (45, 46). Since these studies did not perform predictor-analyses, these data were not applicable for this review.

The third hypothesis assumed that physical and psychological conditions such as food sensation, executive functions, strain and dissatisfaction or sex and diseases predict BWL, whereas for BWLM only food sensation and the executive functions self-monitoring and self-regulation are positive predictors. Of these factors, only food sensation (27) and self-regulation were predictors (BWL). Impulsivity was positive predictive (31), attention shifting and inattention were found as negative or non-predictive (20, 31, 40), whereas other factors of self-regulation such as a stop signal task and sensitivity to reward were non-predictive (23, 25). Interestingly, food sensation and self-regulation (BWLM), sex and diseases (BWL) that were important for adults, did not predict BWL or BWLM in children (22, 23, 25, 35, 37, 39, 40, 42, 43). Nevertheless, sex could play a role as a moderator or mediator considering that sex made a difference in predictors such as C-peptide concentration and telomere length (24, 26). Regarding diseases, it must be considered that children do not have as many diseases as adults and their diseases differ from those found in adults. So far, psychological strain/dissatisfaction was none-predictive. Additionally, research about problem-solving ability (BWL) and self-monitoring (BWLM) is missing, with the literature instead assessing many other predictors (Supplementary Material 3 and Supplementary Material 4). In particular, genes and hormones were fields with many predictors.

As a fourth hypothesis, it was assumed that compliance and compliance influencing factors do predict BWL and BWLM. In line with our hypothesis, BWL was positively predicted by the child's motivation (22). Other important positive predictors for BWL were adherence to treatment and compliance to adjuvant therapies (22). This emphasizes the crucial role of compliance for treatment success. The influence of weight loss history on BWL and self-efficacy on BWLM was not investigated as predictors in children.

### Strengths and Limitations

This systematic review involved a broad search of predictors of BWL and BWLM in children and adolescents to ensure that all possible literature would be found. Although, the amount of pre-post investigations on BWL *per se* is large, most studies perform no predictor analyses. One important reason may be due to experimental designs with different aims and small sample sizes not allowing for predictor analyses. Thus, the amount and quality of eligible studies for this review was only moderate, and research aims were heterogeneous making it very difficult to draw conclusions. Especially for BWLM, the amount of studies was small. The included studies only investigated children between 9 and 14 years, with a median age of 12.5 years. There is a huge gap in the literature for information on younger children and adolescents. Thus, most investigated variables have been parent and not self-reports. This may also explain why studies did not focus on energy intake, in contrast to the adults' sector. A few predictors showed sex specifications, but most predictors were not investigated for this factor. Moreover, age, initial BMI and treatment approach were potential predictors in all studies, but were mostly not reported. It is likely that more non-significant results are hidden in those non-reports, giving the impression, that those predictors have no influence. Regarding diseases, it must be considered that only a few diseases with importance for children were investigated. This might be partly explained by our eligibly criteria that excluded papers that focus solely on cohorts with obesity and diseases. Furthermore, changes in body weight composition were not considered as outcome in this systematic review because this would have limited the number of eligible studies dramatically. Since body composition is an important marker for health status, we recommend that in future predictor analysis, body composition (body fat mass, body fat mass index) (4–6) should be considered equally to body weight status. A clear strength of this review is the comparison of our findings with existing research regarding adults BWL and BWLM. The novel approach to summarize the very heterogenic predictors in several models gives a picture of the current research situation regarding longitudinal studies. The clear visualization provides an overview and outlines research gaps. Future research should investigate longitudinally the predictors of children‘s weight loss and weight loss maintenance along with body composition and considering different ages. Due to the missing literature, final conclusions are difficult to draw for children, different to the situation in adults where more literature is available (11).

## Conclusion

In this review the predictors for BWL and BWLM after a behavioral BWL intervention in children were identified and important research gaps outlined. The expected key predictor in research for adults behavior was hardly investigated in children. For both BWL and BWLM, physiology was the most examined cluster and the topic with the most identified significant predictors, in particular for genetics (BWL) and blood parameters (BWLM). Furthermore, several factors from the cluster psychology were investigated and predicted BWL and BWLM. The cluster environment, which was highlighted in most intervention guidelines was neglected in studies regarding BWLM and hardly investigated in studies with BWL. Overall, more than 80% of the investigated predictive factors were only investigated once and studies on predictive factors in young children, adolescents and for BWLM were scarce.

## Data Availability Statement

The original contributions presented in the study are included in the article/Supplementary Material, further inquiries can be directed to the corresponding author.

## Author Contributions

AW, LN, and IM: conceptualization and writing-original draft preparation. AW, LN, and KZ: methodology. AW, LN, IM, KZ, SE, FJ, and SZ: discussion and writing-review and editing. AW and LN: visualization and project administration. IM: supervision. All authors have read and agreed to the published version of the manuscript.

## Funding

The current project was funded by the Innovation Committee of the German Joint Federal Committee (G-BA) with the funding number 01NVF18013 (STARKIDS). The authors acknowledge support by the Deutsche Forschungsgemeinschaft and the Open Access Publishing Fund of Tübingen University.

## Conflict of Interest

The authors declare that the research was conducted in the absence of any commercial or financial relationships that could be construed as a potential conflict of interest.

## Publisher's Note

All claims expressed in this article are solely those of the authors and do not necessarily represent those of their affiliated organizations, or those of the publisher, the editors and the reviewers. Any product that may be evaluated in this article, or claim that may be made by its manufacturer, is not guaranteed or endorsed by the publisher.

## Abbreviations

BWL: body weight loss
BWLM: body weight loss maintenance
BMI: body mass index.
