# Supplementary material for: Predictors of Weight Loss and Weight Loss Maintenance in Children and Adolescents With Obesity After Behavioral Weight Loss Intervention
Source: Front Public Health. 2022 Mar 25;10:813822. doi: 10.3389/fpubh.2022.813822 (PMC8989956; doi:10.3389/fpubh.2022.813822)
Supplement: Supplementary file 1 [file Data_Sheet_1.pdf]

Review article

Predictors of weight loss and weight-loss maintenance in children and adolescents with obesity after behavioural weight loss intervention

Alisa Weiland [1†], Lena Kasemann [1†], Stephan Zipfel [1], Stefan Ehehalt [2], Katrin Ziser [1], Florian Junne[1,3], Isabelle Mack[1]*

[1] Department of Psychosomatic Medicine and Psychotherapy, University Medical Hospital,

72072 Tübingen, Germany; [2] Public Health Department of Stuttgart, Stuttgart, Germany; [3] Department of Psychosomatic Medicine and Psychotherapy, University Hospital Magdeburg, Otto von Guericke University Magdeburg

† Both authors contributed equally to this work.

***** Correspondence: isabelle.mack@uni-tuebingen.de; Tel.: +49-7071-2985614; Fax: +49-7071-294382

Supporting Information

Supplement 1: Study-Characterization

Study	Intervention	Sample Size and Characterizatio		Predictors			QAT
Author (year)	Study type;<br>Country;<br>Intervention;<br>Lengths;<br>Follow Up;<br>In-/Outpatient;<br>Parents-involvement	n; Age (SD);<br>Sex (%), BMI [z-score] (SD)		Topic, direction (↑, ↓ or ↔), reported statistics (outcome, method)			
Augustijn et al. (2018)	NRCT;<br>B;<br>Diet-Sport-Behaviour;<br>5 months (6-10 months);<br>No follow up;<br>Outpatient;<br>No parents involved	**I:**<br><br><br><br>**C:**	**BL:** n=32; age: 9.6 (1.1); 56% ♀; BMI: 2.7 (0.3)<br>**T1:** n=30; age: 9.9 (1.2); 60% ♀, BMI: 2.0 (0.4)<br>**BL:** n=32; age: 9.6 (1.2); 44% ♀; BMI: 0.1 (0.5)<br>**T1:** n=25; age: 9.9 (1.2); 28% ♀; BMI: 0.1 (0.5)	Hierarchical regression, controlled for age and sex			**4**
				Motor competence and executive functions	↑	F=4.74, p=.003, Effect size: 32.3%	
				Manual dexterity	↔	/	
				Ball skills (after inclusion of the outliers)	↑ (↑)	N.R., Effect size: 16.3% (p FDR=.084)	
				Static and dynamic balance skills	↔	/	
				Attention shifting (intra-extra dimensional shift): IED pre-ED errors, IED total trials (adjusted)	↔	/	
				Updating abilities and inhibition control (rapid visual information processing): RVP total hits, RVP total false alarms	↔	/	
				Planning and decision making (Stockings of Cambridge): SOC problem solved in minimum moves, SOC mean moves, SOC mean initial thinking time (ms), SOC mean subsequent thinking time (ms)	↔	/	
Boutelle et al. (2019)	NRCT;<br>U.S.;<br>Diet-Sport-Behaviour;<br>6 months;<br>6 & 12 months follow up;<br>Outpatient;<br>Parents involved	**I:**<br><br>ISR:<br><br>IFR:<br><br>IEE:<br><br><br>ISR:<br><br>IFR:	**BL:** n=150; age: 10.41 (1.27); 67% ♀; BMI: 2.0 (0.3)<br>**BL:** n=71; age: 10.59 (1.32); 63% ♀; BMI: 2.0<br>**BL:** n=52; age: 10.37 (1.18); 69% ♀; BMI: 1.9<br>**BL:** n=27; age: 10.03 (1.25); 70% ♀; BMI: 2.0<br><br>**T1:** n=71; age: 10.59 (1.32); 63% ♀; BMI: 1.77<br>**T1:** n=52; age: 10.37 (1.18);	Linear mixed-effect models			3
				Phenotype appetitive trajectory group	↔	/	
				Phenotype High Satiety Responsive group (M)	$\uparrow^{M}$	N.R., p<.05	

		 IEE:<br><br>ISR:<br><br>IFR:<br><br>IEE:<br><br>ISR:<br><br>IFR:<br><br>IEE:	69% ♀; BMI: 1.75<br>**T1:** n=27; age: 10.03 (1.25);<br>70% ♀; BMI: 1.78<br><br>**M1:** n=N.R.; age: N.R.;<br>N.R.; BMI: 1.74<br>**M1:** n=N.R.; age: N.R.;<br>N.R.; BMI: 1.85<br>**M1:** n=N.R.; age: N.R.;<br>N.R.; BMI: 1.85<br><br>**M2:** n=N.R.; age: N.R.;<br>N.R.; BMI: 1.74<br>**M2:** n=N.R.; age: N.R.;<br>N.R.; BMI: 1.88<br>**M2:** n=N.R.; age: N.R.;<br>N.R.; BMI: 1.87<br>**SR=high satiety responsive group, FR=high food responsive group, EE=high emotional eating group**				
Celi et al. (2003)	BA;<br>I;<br>Diet-Sport-Behaviour;<br>12 months;<br>12 months follow up;<br>Outpatient;<br>No parents involved	**I:**	**BL:** n=172; age: N.R.;<br>52% ♀; BMI: 1.94 (0.55)<br>**T1:** n=172; age: N.R.;<br>52% ♀; BMI: 1.5 (0.59)<br>**M1:** n=172; age: N.R.;<br>52% ♀; BMI: 1.48 (0.68)	Univariate regression analysis			3
				Age	$\leftrightarrow^{M}$	/	
				Initial BMI z-score	$\leftrightarrow^{M}$	/	
				Sex distribution (%)	$\leftrightarrow^{M}$	/	
				Serum leptin concentration (BL)	$\leftrightarrow^{M}$	/	
				Insulin	$\leftrightarrow^{M}$	/	
				Blood lipids	$\leftrightarrow^{M}$	/	
				Blood pressure	$\leftrightarrow^{M}$	/	
				BMI parents	$\leftrightarrow^{M}$	/	
				Multivariate regression model, odds			
				Sex	$\leftrightarrow^{M}$	/	
				Pubertal stage	$\downarrow^{M}$	OR 0.78; 95% CI[0.63-0.96]	
				BMI change, after I	$\leftrightarrow^{M}$	/	
				Serum leptin concentration after I	$\uparrow^{M}$	OR 1.08; 95% CI[1.04-1.01]	
				Serum leptin concentration change	$\downarrow^{M}$	OR 0.48; 95% CI[0.25-0.92]	
Dubuisson et al. (2012)	BA;<br>B;<br>Diet-Sport-Behaviour;	**I:**	**BL:** n=144; age: 10.5 (3.1);<br>59% ♀; BMI: 2.73 (0.6)<br>**T1:** n=144; age: 10.5 (3.1);<br>59% ♀; BMI: 2.59 (0.6)	Nonlinear regression by P splines			2
				Daily intake: fruits, juice, vegetables, soup, cookies (BL)	$\leftrightarrow$	/	
				Daily intake: water (BL)	$\uparrow$	N.R., p=.046	
				Daily intake: soda (BL)	$\downarrow$	N.R., p<.001	

	9 months (26 months);<br>No follow up;<br>Outpatient;<br>No parents involved			Number of visits, intervention lengths	↔	/	
				Adherence to treatment	↑	N.R., p<.001	
				Compliance to adjuvant therapies	↑	N.R., p<.001	
				Age	↔	/	
				Physical health: asthma, gestational diabetes, mental health, bad quality of sleep	↔	/	
				Growth	↔	/	
				Eating behaviour: eating breakfast every day, 2 hot meals a day, snacker, large portions, eating disorder pathology	↔	/	
				Familial encouragement to the project	↑	N.R., p=.004	
				Familial encouragement to leisure activities	↔	/	
				History: birth weight (>4000g), breastfeeding>5 months	↔	/	
				Initial BMI	↔	/	
				Motivation	↑	N.R., p<.001	
				Obesity in family	↔	/	
				Only child	↑	N.R., p=.026	
				Parents presence after school, dual household	↔	/	
				Physical activity	↑	N.R., p=.037	
				Delayed Puberty	↓	N.R., p=.046	
				Sex	↔	/	
				Social integration	↔	/	
				Delta weight change	↑	N.R.	
				Recent weight gain	↔	/	
Eichen et al. (2018)	BA;<br>U.S.;<br>Diet-Sport-Behaviour;<br>3 & 6 months;<br>6 & 18 months follow up;<br>Outpatient;<br>Parents involved	**I:**	**BL:** n=150; age: 10.4 (1.3); 66.7% ♀; BMI: 2.0 (0.3)<br>**T1:** n=125.; age: N.R.; N.R.; BMI: 1.85 (0.39)<br>**T2:** n=124.; age: N.R.; N.R., BMI: 1.73 (0.44)<br>**M1:** n=128.; age: N.R.; N.R.; BMI: 1.79 (0.46)<br>**M2:** n=131.; age: N.R.; N.R.; BMI: 1.81 (0.46)	Linear mixed effect models			5
				Planning and decision making: Child Wisconsin Card Sorting Test: Perseverative Errors	↔<br>$\leftrightarrow^{M1}$<br>$\uparrow^{M2}$	/<br>/<br>t=3.40, p<.001, r=.11	
				Parent Planning and decision making: Wisconsin Card Sorting Test: Perseverative Errors	↔	/	
				Parent executive functions	↔<br>$\leftrightarrow^{M1}$<br>$\leftrightarrow^{M2}$	/<br>/<br>/	
				Child digit span	↔<br>$\leftrightarrow^{M1}$<br>$\leftrightarrow^{M2}$	/<br>/<br>/	
				Self-regulation ability: Child Stop Signal Task failures	↔<br>$\leftrightarrow^{M1}$<br>$\leftrightarrow^{M2}$	/<br>/<br>/	

				Executive functions ↔<br><br>Executive functions ↔ ↑			
García-Calzón et al. (2014)	BA;<br>E;<br>Diet-Sport-Behaviour;<br>2 months;<br>6 months follow up;<br>Inpatient;<br>No parents involved	I:<br><br>IB:<br><br>IG:<br><br><br><br>IB:<br><br>IG:<br><br><br><br>IB:<br><br>IG:	**BL:** n=74; age: 14.3 (1.0);<br>51% ♀; BMI: 4.8 (1.8)<br>**BL:** n=36; age: 14.3 (0.9);<br>0% ♀; BMI: 5.0 (1.6.)<br>**BL:** n=38; age: 14.4 (1.1);<br>100% ♀; BMI: 4.6 (2.0)<br><br>**T1:** n=36; age: N.R.;<br>0% ♀; BMI: 4.1(1.7.)<br>**T1:** n=38; age: N.R.;<br>100% ♀; BMI: 3.9 (1.7)<br><br>**M1:** n=36; age: N.R.;<br>0% ♀; BMI: 3.2 (1.6.)<br>**M1:** n=38; age: N.R.;<br>100% ♀; BMI: 3.9 (1.9)<br>B=boys, G=girls	Repeated measures ANOVA<br>Telomere lengths (BL) boys<br><br>Telomere lengths (BL) girls<br><br>Multivariable linear regression<br>Telomere lengths (BL) boys (median split)	 ↑<br>$\uparrow^{M}$<br>↔<br>$\leftrightarrow^{M}$<br><br>↑<br>$\uparrow^{M}$	 B=-0.22, p=.010<br>B=-0.47, p=.005<br>/<br>/<br><br>B=-2.85, p=.001<br>B=-3.99, p=.014	3
Halberstadt et al. (2013)	NRCT;<br>NL;<br>Diet-Sport-Behaviour;<br>12 months;<br>12 months follow up;<br>Outpatient (including 2 or 6 months in);<br>Parents involved	I:<br><br>IC:	**BL:** n=120; age: 14.8 (2.4);<br>68% ♀; BMI: 3.41 (0.38)<br>**BL:** n=74; age: 14.9 (2.3);<br>70% ♀; BMI: 3.44 (0.36)<br>**T1:** n=74; age: 14.9 (2.3);<br>70% ♀; BMI: 3.03 (0.63)<br>**M1:** n=74; age: 14.9 (2.3);<br>70% ♀; BMI: 3.18 (0.67)<br>C=group with complete data	Linear regression models<br>Self-regulation ability: inhibitory control (Stop-Signal task); sensitivity to reward after intervention<br>Self-regulation ability: inhibitory control (Stop-Signal task); sensitivity to reward after baseline	 ↔<br>↔<br>↔<br>$\leftrightarrow^{M}$	 /<br>/<br>/<br>/	3
Kloppenborg et al. (2017)	NRCT;<br>DK;<br>Diet-Sport-Behaviour;<br>12 months;<br>No follow up;<br>Outpatient;<br>No parents involved	I:<br><br>IC:<br><br><br><br><br><br><br><br>IB:<br><br>IG:	**BL:** n=752; N.R.;<br>56% ♀; BMI: 2.91<br>**BL:** n=569; N.R.;<br>41% ♀; BMI: 2.94 (0.68.)<br>**T1:** n=569; N.R.;<br>41% ♀; BMI: 2.63 (0.82.)<br>**BL:** n=569; N.R.;<br>41% ♀; BMI: 2.94<br><br>**BL:** n=333; age: 11.58;<br>0% ♀; BMI: 3.16 (N.R.<br>**BL:** n=419; age: 11.18;<br>100% ♀; BMI: 2.75	Linear regressions adjusted for treatment duration, age and baseline concentrations<br>Hormone Concentrations (BL): FPG, HbA1c, Insulin, HOMA2-IS, HOMA2-B<br>Serum C-Peptide (BL) boys<br>Serum C-Peptide (BL) girls	 ↔<br><br>↔<br>↑	 /<br><br>/<br>95%CI [0.01-0.1], p=.020	4

			C= group with complete data, B=boys, G=girls				
Mölbert et al. (2016)	BA;<br>D;<br>Diet-Sport-Behaviour;<br>1 month;<br>No follow up;<br>Inpatient;<br>No parents involved	**I:**<br><br><br><br>**C:**	**BL:** n=60; age: 0.0 (0.0); 53% ♀; BMI: 2.51 (0.6)<br>**T1:** n=53; age: 0.0 (0.0); 57% ♀, BMI: 2.3 (0.6)<br>**BL:** n=27; age: 0.0 (0.0); 44% ♀; BMI: -0.2 (0.6)	Spearman correlation			4.75
				Body widths (spine, hip, thigh, upper arm)	↔	/	
				Body depths (abdomen, buttocks)	↔	/	
				Body size estimation	↔	/	
				Tactile size estimation	↔	/	
				Heartbeat detection	↔	/	
				Concerns about body weight and shape	↔	/	
Moleres et al. (2014)	BA;<br>E;<br>Diet-Sport-Behaviour;<br>2.5 months;<br>No follow up;<br>Outpatient;<br>No parents involved	**I:**	**BL:** n=199; age: 14.5 (1.13); 60% ♀; BMI: 4.5 (2.82)<br><br>**T1:** n=199; age: N.R; 60% ♀, BMI: 3.8 (1.41)	Multivariant regression models			
				APOA1: rs670	↑	R^2=0.213, B=-0.234, SEM=0.05, p<.001 (6.03x1-6)	
				CETP: rs1800777	↑	R^2=0.174, B=-0.62, SEM=0.17, p<.001 (4.8x10-4)	
				APOA1 + CETP	↑	R^2=0.241, B=-0.244, SEM=0.05, p<.001 (2.3x10-7)	
				FTO: rs9939609	↔	/	
				APOA5: rs662799	↔	/	
Moleres et al. (2012)	BA;<br>E;<br>Diet-Sport-Behaviour;<br>3 months;<br>No follow up;<br>Outpatient;<br>No parents involved	**I:**	**BL:** n=168; age: 14.6 (0.09); 62% ♀; BMI: 4.5 (0.2)<br><br>**T1:** n=168; age: N.R.; 62% ♀, BMI: 3.7 (0.1)	Multiple linear regression analysis			4.8
				Genetic predisposition score	↑	Allele effect size=-0.26, SE=0.02, p<.001	
				FTO: rs9939609	↑	Allele effect size=-0.18, SE=0.05, p=.018	
				TMEM18: rs7561317	↑	Allele effect size=-0.212, SE=0.06, p=.005	
				MC4R: rs17782313	↔	/	
				PPARG: rs1801282	↔	/	
				IL6: rs1800795	↔	/	
				ADIPOQ: rs822395, rs2241766, rs1501299	↔	/	
Murer et al. (2011)	BA;<br>D;<br>Diet-Sport-Behaviour;<br>2 months;<br>6 & 12 months follow up;<br>Inpatient;<br>No parents involved	**I:**	**BL:** n=203; age: 14.1 (2.0); 44% ♀; BMI: 2.3 (0.3)<br><br>**T1:** n=203; age: 14.3 (2.0); 44% ♀, BMI: 1.9 (0.4)<br><br>**M1:** n=139; age: N.R.; N.R. ♀; BMI: 1.8 (0.5)<br><br>**M2:** n=100; age: N.R.;	Multivariant regression models			5
				Leptin level (BL)	↓<br>↓^{M1}<br>↔^{M2}	ß=-0.51, p<.001<br>ß=-0.24, p<.008<br>/	
				Leptin Level decrease during I	↔<br>↑^{M1}<br>↔^{M2}	/<br>ß=0.21, p=.017<br>/	

			N.R. ♀; BMI: 1.8 (0.5)				
Pauli-Pott et al. (2010)	BA;<br>D;<br>Diet-Sport-Behaviour;<br>12 months;<br>No follow up;<br>Outpatient;<br>No parents involved	**I:**	**BL:** n=111; age: 11.1 (2.0);<br>57% ♀; BMI: 2.43 (0.4)<br><br>**T1:** n=95; age: N.R.;<br>N.R. ♀; BMI: 2.13 (N.R.)	Logistic regression analysis			5
				Inattention	↔	/	
				Impulsivity	↑	X2=3.88, R^2=0.04, p<.049	
Pott et al. (2010)	BA;<br>D;<br>Diet-Sport-Behaviour;<br>12 months;<br>No follow up;<br>Outpatient;<br>No parents involved	**I:**<br><br>**Ir:**<br><br>**Inr:**	**BL:** n=136; age: 11.5 (1.85);<br>54% ♀; BMI: 2.46 (0.4)<br>**BL:** n=80; age: N.R.;<br>N.R. ♀; BMI: N.R.<br>**BL:** n=56); age: N.R.;<br>N.R. ♀; BMI: N.R.<br>(dropout=19, less weight loss=37<br>**T1:** n=117; age: N.R.;<br>N.R. ♀; BMI: 2.16 (N.R.)<br>r=responder, nr=non-responder	Hierarchical linear regression analysis			4
				Depression, depressive symptoms (BL)	↔	/	
Pott et al. (2009)	BA,<br>D;<br>Diet-Sport-Behaviour;<br>12 months;<br>No follow up;<br>Outpatient;<br>No parents involved	**I:**	**BL:** n=111; age: 11.5 (1.85);<br>57% ♀; BMI: 2.43 (0.44)<br><br>**T1:** n=95; age: N.R.;<br>N.R. ♀, BMI: 2.13 (N.R.)	Logistic regression analysis			4
				Obese siblings	↓	R^2=0.21, p<.001	
				Age	↔	/	
				Maternal attachment attitude	↔	/	
				Maternal depression	↔	/	
Rendo-Urteaga et al. (2014)	BA;<br>E;<br>Diet-Sport-Behaviour;<br>2.5 months;<br>No follow up;<br>Outpatient;<br>No parents involved	**I:**<br><br>**Ihr:**<br><br>**Ilr:**<br><br>**Ihr:**<br><br>**Ilr:**	**BL:** n=12; age: 13 (N.R.);<br>0% ♀; BMI: 3.10 (N.R.)<br>**BL:** n=6; age: 13.17 (2.4);<br>0% ♀; BMI: 3.10 (0.54)<br>**BL:** n=6; age: 12.83 (3.91);<br>0% ♀; BMI: 4.08 (0.43)<br><br>**T1:** n=6; age: 13.17 (2.4);<br>0% ♀; BMI: 2.46 (0.48)<br>**T1:** n=6; age: 12.83 (3.91);<br>0% ♀; BMI: 4.01 (0.46)	Linear regression model			5
				PBMC low expression of: TFPI	↑	B=0.50, CI (95%)=0.11 – 0.88, p=.017	
				PBMC low expression of: LEPR	↑	B=0.59, CI (95%)=0.02 – 1.16, p=.045	
				PBMC low expression of: LTBP1	↑	B=0.57, CI (95%)=0.12 – 1.01, p=.019	
				PBMC low expression of: MMRN1	↑	B=0.53, CI (95%)=0.13 – 0.92, p=.016	
				PBMC low expression of: PKHD1L1	↑	B=0.53, CI (95%)=0.11 – 0.95, p=.019	

			hr=high responder, lr=low responder	PBMC low expression of: SIRPB1<br>PBMC low expression of: EFG, JAM3; SELP, IGKC	↑<br>↔	B=0.28, CI (95%)=0.10 – 0.46, p=.006<br>/	
Röbel et al. (2013)	BA;<br>D;<br>Diet-Sport-Behaviour;<br>6-24 months;<br>6-36 months follow up;<br>N.R.;<br>No parents involved	I:	**BL:** n=12,305; age: N.R.; N.R. ♀; BMI: 2.44 (0.54)<br><br>**T1:** n=12,305; age: N.R.; N.R. ♀, BMI: N.R.<br><br>**M:** n=12,305; age: N.R.; N.R. ♀, BMI: N.R.	Linear regression model (ANCOVA)<br>Age<br>Sex<br>Initial BMI<br>Treatment duration<br>Low/ no social risk factors	 ↔<br>↔<br>↔<br>↔<br>↑	 /<br>/<br>/<br>/<br>p<.001	4
Roth et al. (2013)	NRCT;<br>D;<br>Sport-Behaviour;<br>12 months;<br>No follow up;<br>Outpatient;<br>No parents involved	I:<br><br><br><br>C:	**BL:** n=451; age: 12; 55% ♀; BMI: 2.4 (0.5)<br>**T1:** n=451; age: N.R.; 55% ♀, BMI: 2.12 (N.R.)<br>**BL:** n=583; age: 25.3; 60% ♀; BMI: 19.1 (1.9)<br>BMI C is BMI, not z-BMI	Linear regression analysis<br>Dopamine receptor gene polymorphism (DRD2)<br>Dopamine receptor gene polymorphism (DRD4)	 ↔<br>↔	 /<br>/	4
Santoro et al. (2007)	BA;<br>I;<br>Diet-Sport-Behaviour;<br>6 & 12 months;<br>No follow up;<br>Outpatient;<br>No parents involved	I:<br>Iw:<br>Ih:<br><br>Iw:<br>Ih:<br><br>Iw:<br>Ih:	**BL:** n=184; age: 9.2 (2.0); 42% ♀; BMI: 3.38<br>**BL:** n=164; age: N.R.; N.R. ♀; BMI: 3.4 (2.2)<br>**BL:** n=20; age: N.R.; N.R. ♀; BMI: 3.1 (2.4)<br><br>**T1:** n=164; age: N.R.; N.R. ♀, BMI: 2.1 (1.4)<br>**T1:** n=20; age: N.R.; N.R. ♀, BMI: 2.5 (1.6)<br><br>**T2:** n=164; age: N.R.; 00% ♀, BMI: 1.7 (1.1)<br>**T2:** n=20; age: N.R.; 00% ♀, BMI: 2.2 (1.9)<br>w=wildtype, h=heterozygote	3-factor repeated measures ANOVA<br>Sex<br>Melanocortin-3 Receptor polymorphism [heterozygote (C17A and G241A)]	 ↔<br>↓	 /<br>N.R., p=.003	2.33
Sauer et al. (2017)	NRCT;<br>D;<br>Diet-Sport-Behaviour;<br>1 month;	I:	**BL:** n=60; age: 13.03 (1.89); 53% ♀; BMI: 2.51 (0.6)<br><br>**T1:** n=53; age: 13.04 (1.85); 57% ♀, BMI: 2.31(N.R.)	Spearman correlation<br>Total taste identification score (BL)<br>Taste ability sour, salty (BL)<br>Linear regression analysis<br>Taste ability sweet (BL)	 ↔<br>↔<br><br>↑	 /<br>/<br><br>ß=0.32, t[50]=2.63, p=.011	5

	No follow up;<br>Inpatient;<br>No parents involved	C:	**BL:** n=27; age: 12.52 (0.94);<br>44% ♀; BMI: -0.2 (0.1)	Taste ability bitter (BL)<br>Sweet and bitter score (BL)	↓<br>↑↓	ß=-0.40, t[50]=-3.29, p=.002<br>R2=0.26, adjusted R^2=0.23, F[2,50]=8.56, p=.001	
Vander et al. (2013)	BA;<br>U.S.;<br>Diet-Sport-Behaviour;<br>1 month;<br>No follow up;<br>Inpatient;<br>No parents involved	I:<br>I+:<br>I-:<br>I+:<br>I-:	**BL:** n=73; age: (13.44);<br>78% ♀; BMI: N.R. (N.R.)<br>**BL:** n=16; age: N.R. (N.R.);<br>N.R. % ♀; BMI: 0.0 (0.0)<br>**BL:** n=55; age: N.R. (N.R.);<br>N.R. ♀; BMI: 0.0 (0.0)<br>**T1:** n=16; age: N.R. (N.R.);<br>N.R. ♀; BMI: 2.02 (N.R)<br>**T1:** n=55; age: N.R. (N.R.);<br>N.R. ♀, BMI: 2.01 (N.R.)<br>"+"=Antibody positiv, „-„=Antibody negativ	Multiple regression analysis<br>Sex<br>Neck circumference, hip<br>Blood lipids<br>Adenovirus 36 antibody positive	 ↔<br>↔<br>↔<br>↔	 /<br>/<br>/<br>/	4
van Egmond-Froehlich et al. (2012)	BA;<br>D;<br>Diet-Sport-Behaviour;<br>12 months;<br>12 months follow up;<br>Outpatient;<br>Parents involved	I:	**BL:** n=394; age: 11.7 (2.0);<br>57% ♀; BMI: 2.32 (0.46)<br><br>**T1:** n=394; age: N.R.;<br>57% ♀, BMI: 2.11 (0.60)<br><br>**M:** n=311; age: N.R.;<br>N.R.% ♀; BMI: 2.14 (0.66)	Univariate general regression model<br>Hyperactivity/ Inattention<br><br><br>Conduct problem<br><br>Eating disinhibition<br><br>Maternal BMI<br><br>Socioeconomic status score (SES)	 ↓^{short}<br>↓^{long}<br>↔^{M}<br>↔<br>↔^{M}<br>↔<br>↔^{M}<br>↔<br>↔^{M}<br>↔	 F=23.7, eta2=0.06, p<.001<br>F=17.7, eta2=0.05, p<.001<br>/<br>/<br>/<br>/<br>/<br>/<br>/<br>/	4
Wolters et al. (2013)	BA;<br>D;<br>Diet-Sport-Behaviour;<br>12 months;<br>12 months follow up;<br>Outpatient;<br>No parents involved	I:	**BL:** n=477; age: 10.6 (2.7);<br>54% ♀; BMI: 2.49 (0.40)<br><br>**T1:** n=477; age: N.R.;<br>54% ♀, BMI: 2.17 (0.53)<br><br>**M1:** n=477.; age: N.R.;<br>54% ♀; BMI: 2.22 (0.59)	Linear regression model<br>High concentration TSH (BL)<br>High concentration fT3 (BL)<br>High concentration fT4 (BL)<br>TSH concentration decrease during I<br>fT3 concentration decrease during I<br>fT4 concentration decrease during I	 ↑<br>↑<br>↔<br>↓^{M}<br>↓^{M}<br>↔^{M}	 r=0.21, p<.001; R2=0.13, p<.001<br>r=0 .22; p<.001; R2=0.11, p=.002<br>/<br>r=-0.11; p=.010; R2=0.04, p=.009<br>r=-0.16, p<.001; R2=0.05, p=.003<br>/	4
Zamrazilova et al. (2015)	BA;<br>CZ;<br>Diet-Sport;<br>1 month;<br>No follow up;<br>Inpatient;<br>No parents involved	I:<br>I+:<br>I-:<br>I+:	**BL:** n=184; age: 14.89 (0.22);<br>100% ♀; BMI: 2.85<br>**BL:** n=65; age: N.R.;<br>100% ♀; BMI: 2.76 (0.09)<br>**BL:** n=119; age: N.R.;<br>100% ♀; BMI: 2.90 (0.07)<br>**T1:** n=65; age: N.R.;<br>100% ♀, BMI: 2.41 (N.R.)	General linear model analysis<br>Adenovirus 36 antibody positive	 ↔	 /	2

		**I**-:	**T1:** n=119; age: N.R.; 100% ♀, BMI: 2.59 (N.R.)<br>“+”=Antibody positiv, „-„=Antibody negativ		

Abbreviations
QAT: Quality Assessment Tool; n: Number; SD: Standard deviation; BMI: Body mass index [z-score] ♀: female; C: Control Group; I: Intervention; BL = Baseline: T = Measurement after Treatment; M = Measurement after Intervention; RCT: Randomized controlled trial; BA: Before-and-after comparison (without control); NRCT: Nonrandomized controlled trial; B = Belgium; U.S.= United States of America; UK = United Kingdom; I = Ital; E = England; DK = Denmark; D = Germany; CH = Switzerland; CZ = Czech Republic; N.R. = not reported

Supplement 2: Risk of Bias (Quality Assessment Tool)

study	sum	representativeness of cohort	drop out bias	validity and reliability	weight measurement	sample size	statistics	comments
Augustijn et al., (2018)	4	0	1	0	1	1	1	col. 1: participation rate at baseline not described<br>col. 3: test-retest correlations for questionnaires not described
Boutelle et al., (2019)	3	0	0	1	0	1	1	col. 1: participation rate at baseline not described, selectiveness of non-response not described
Celi et al., (2003)	3	0	0	1	0	1	1	col. 1: participation rate at baseline not described, selectiveness of non-response not described
Dubuisson et al., (2012)	2	N.R.	0	0	1	0	1	col. 1: not appropriate (retrospective analysis)
Eichen et al., (2018)	5	0	1	1	1	1	1	col. 1: participation rate at baseline not described, selectiveness of non-response not described
García-Calzón et al., (2014)	3	0	0	1	1	0	1	col. 1: participation rate at baseline not described, selectiveness of non-response not described
Halberstadt et al., (2017)	3	0	0	0	1	1	1	col. 1: participation rate at baseline not described, selectiveness of non-response not described<br>col. 3: test-retest correlations for questionnaires not described
Kloppenborg et al., (2017)	4	0	0	1	1	1	1	col. 1: participation rate at baseline not described, selectiveness of non-response not described
Mölbert et al 2016	4,75	0	1	0,75	1	1	1	col. 1: participation rate at baseline not described, selectiveness of non-response not described
Moleres et al 2014	4,8	0	1	0,8	1	1	1	col. 1: participation rate at baseline not described, selectiveness of non-response not described
Moleres et al 2012	5	0	1	1	1	1	1	col. 1: participation rate at baseline not described, selectiveness of non-response not described

Murer et al 2011	5	0	1	1	1	1	1	col. 1: participation rate at baseline not described, selectiveness of non-response not described
Pauli-Pott et al 2010	4	0	1	0	1	1	1	col. 1: participation rate at baseline not described, selectiveness of non-response not described
Pott et al 2009	4	0	1	0	1	1	1	col. 1: participation rate at baseline not described, selectiveness of non-response not described
Pott et al 2010	4	0	1	0	1	1	1	col. 1: participation rate at baseline not described, selectiveness of non-response not described<br>col. 3: test-retest correlations for questionnaires not described
Rendo-Urteaga et al 2014	5	0	1	1	1	1	1	col. 1: participation rate at baseline < 80 % und selectiveness of non-response not described
Röbl et al 2013	5	1	1	0	1	1	1	
Roth et al 2013	4	0	0	1	1	1	1	col. 1: participation rate at baseline not described, selectiveness of non-response not described
Santoro et al 2007	2,33	0	0	0,33	1	0	1	
Sauer et al 2016	5	0	1	1	1	1	1	col. 1: participation rate at baseline not described, selectiveness of non-response not described
Vander Wal et al 2012	4	0	0	1	1	1	1	col. 1: participation rate at baseline not described, selectiveness of non-response not described
Van Egmond-Froehlich et al 2012	4	0	0	1	1	1	1	col. 1: participation rate at baseline < 80 % und selectiveness of non-response not described
Wolters B. et al 2012	4	0	0	1	1	1	1	col. 1: participation rate at baseline not described, selectiveness of non-response not described
Zamrazilová et al 2015	2	0	0	1	0	1	0	col. 1: participation rate at baseline not described, selectiveness of non-response not described
**Total**		**1**	**12**	**15,88**	**21**	**21**	**23**	

Supplement 3: Overview Predictors BWL

Overview Predictors Body Weight Loss (BWL)							
Augustijn et al. (2018), Boutelle et al. (2019), Dubuisson et al. (2012), Eichen et al. (2018), García-Calzón et al. (2014), Halberstadt et al. (2017), Kloppenborg et al. (2017), Mölbert et al. (2016), Moleres et al. (2012), (2014), Murer et al. (2011), Pauli-Pott et al. (2010), Pott et al. (2009), Rendo-Urteaga et al. (2014), Röbl et al. (2013), Roth et al. (2013), Santoro et al. (2007), Sauer et al. (2017), Van Egmond-Frohlich et al. (2012), Van der Wal et al. (2012), Wolters et al. (2012), Zamrazilová et al. (2015)							
**Predictor**	**Measurement**	**Significant prediction of BWL**			**N.R.**	**N.A.**	**n**
		positive ($\uparrow$)	negative ($\downarrow$)	none ($\leftrightarrow$)			
**Physiology (n studies =17)** Dubuisson et al. (2012), García-Calzón et al. (2014), Kloppenborg et al. (2017), Mölbert et al. (2016), Moleres et al. (2012), (2014), Murer et al. (2011), Pott et al. (2009), Rendo-Urteaga et al. (2014), Röbl et al. (2013), Roth et al. (2013), Santoro et al. (2007), Sauer et al. (2017), Van Egmond-Frohlich et al. (2012), Van der Wal et al. (2012), Wolters et al. (2012), Zamrazilová et al. (2015)							
**Demographics (n studies=7)** Dubuisson et al. (2012), Mölbert et al. (2016), Pott et al. (2009), Röbl et al. (2013), Santoro et al. (2007), Vander Wal et al. (2012), Van Egmond-Frohlich et al. (2012)							
Sex	Interview/ Questionnaire			$\leftrightarrow^{4}$	19	0	**4**
Age	Interview/ Questionnaire			$\leftrightarrow^{3}$	20	0	**3**
Delayed puberty	Tanner stage		$\downarrow^{1}$		0	22	**1**
Birthweight > 4000g	Homemade Questionnaire			$\leftrightarrow^{1}$	0	22	**1**
BMI z-core	measurement at BL			$\leftrightarrow^{2}$	21	0	**2**
Body size	Growth, body widths (spine, hip, thigh, upper arm), body depths (abdomen, buttocks), neck and hip circumferences			$\leftrightarrow^{9}$	20	0	**3**
Socioeconomic status	SES, low or no social risk factors	$\uparrow^{1}$	$\downarrow^{1}$		0	21	**2**
**Diseases (n studies=3)** Dubuisson et al. (2012), Van der Wal et al. (2012), Zamrazilová et al. (2015)							
Gestational diabetes	Self-report/ Questionnaire			$\leftrightarrow^{1}$	0	22	**1**
Asthma	Self-report/ Questionnaire			$\leftrightarrow^{1}$	0	22	**1**
Andenovirus 36	Antibody positive			$\leftrightarrow^{2}$	0	21	**2**
**Blood parameters (n studies=4)** Kloppenborg et al. (2018), Murer et al. (2011), Vander Wal et al. (2012), Wolters et al. (2012)							
C-peptide boys	BL blood measurement			$\leftrightarrow^{1}$			
C-peptide girls	BL blood measurement	$\uparrow^{1}$			0	22	**1**
Insulin level	BL blood measurement			$\leftrightarrow^{1}$	1	21	**1**
Glucose metabolism	HbA1c BL concentration, impaired fasting glucose, HOMA2-B, HOMA2-IS			$\leftrightarrow^{4}$	0	22	**1**
Blood lipids	Blood measurements			$\leftrightarrow^{1}$	0	22	**1**
BL leptin level	Blood measurements		$\downarrow^{1}$		0	22	**1**
TSH concentration	Blood measurements	$\uparrow^{1}$			0	22	**1**
fT3 concentration	Blood measurements	$\uparrow^{1}$			0	22	**1**
fT4 concentration	Blood measurements			$\leftrightarrow^{1}$	0	22	**1**
**Genetics (n studies=6)** García-Calzón et al. (2014), Moleres et al. (2012), (2014), Rendo-Urteaga et al. (2014), Roth et al. (2013), Santaro et al. (2007)							
Dopamine receptor gene polymorphism (DRD2, DRD4)	DNA extraction and genotyping of blood samples			$\leftrightarrow^{2}$	0	22	**1**
PBMC low expression of: EGF, JAM3, SELP, IGKC)	Gene expression profile analysis of peripheral blood mononuclear cells			$\leftrightarrow^{4}$	0	22	**1**
rs17782313 MC4R	DNA-extraction/genotyping from the buffy coat fraction (BCF)			$\leftrightarrow^{1}$	0	22	**1**
rs1801282 PPARG	DNA-extraction/genotyping from BCF			$\leftrightarrow^{1}$	0	22	**1**
rs1800795 IL6	DNA-extraction/genotyping from BCF			$\leftrightarrow^{1}$	0	22	**1**
rs822395, rs2241766, rs1501299, ADIPOQ	DNA-extraction/genotyping from BCF			$\leftrightarrow^{3}$	0	22	**1**

rs9939609 of FTO	DNA-extraction/genotyping from BCF	↑[1]		↔[1]	0	22	**2**
rs662799 of APOA5	DNA-extraction/genotyping from BCF			↔[1]	0	22	**1**
Telomere length level BL girls	Quantitative real-time polymerase chain reaction			↔[1]	0	22	**1**
Telomere length level BL boys	Quantitative real-time polymerase chain reaction, mean and median split	↑[2]			0	22	**1**
PBMC low expression of: TFPI, LEPR, LTBP1, MMRN1, PKHD1L1, SIRPB1	Analysis of gene expression profile of peripheral blood mononuclear cells	↑[6]			0	22	**1**
Genetic Predisposition Score (GPS)	Constricted by summing the risk alleles across the 9 investigated single-nucleotide polymorphisms	↑[1]			0	22	**1**
rs7561317 (Gene: TMEM18)	DNA-extraction/genotyping from BCF	↑[1]			0	22	**1**
APOA1 (rs670) and/or CETP (rs1800777)	DNA-extraction/genotyping from BCF	↑[3]			0	22	**1**
Melanocortin-3 Receptor polymorphism [heterozygot (C17A and G241A)]	Analysis of genomic DNA from nucleated white blood cells		↓[1]		0	22	**1**
**Taste (n studies=1)** Sauer et al. (2016)							
Taste	Standardized taste identification test at BL: sour, salty, total taste score			↔[3]	0	22	**1**
Taste bitter	Standardized taste identification test at BL, bitter score BL		↓[2]		0	22	**1**
Taste sweet	Standardized taste identification test at BL, sweet score BL	↑[2]			0	22	**1**
**Behaviour (n studies=3)** Augustijn et al. (2018), Boutelle et al. (2019), Dubuisson et al. (2012)							
**Eating behaviour (n studies=2)** Boutelle et al. (2019), Dubuisson et al. (2012)							
Daily intake: fruit, juice, vegetable, soup, cookies	Homemade Questionnaire			↔[5]	0	22	**1**
Daily water intake	Homemade Questionnaire	↑[1]			0	22	**1**
Daily soda intake	Homemade Questionnaire		↓[1]		0	22	**1**
Eating behaviour	Homade Questionnaire: breakfast every day, 2 hot meals a day, snacker, large portions; Phenotype appetitive trajectory group: Satiety vs. Emotional vs. Food based (Child eating Behaviour Questionnaire, Emotional Eating Scale for Children, Eating in the Absence of Hunger for Children)			↔[2]	0	20	**2**
**Energy expenditure (n studies=2)** Augustijn et al. (2018), Dubuisson et al. (2012)							
Manual dexterity	Movement Assessment Battery for Children, Second Edition (MABC-2)			↔[1]	0	22	**1**
Ball skills	MABC-2	↑[2]			0	22	**1**
Preexisting physical activity	Homemade Questionnaire	↑[1]			0	22	**1**
Balance skills (static/dynamic)	MABC-2			↔[2]	0	22	**1**
Motor competence and Executive functions	Sumscore	↑[1]			0	22	**1**

**Weight history (n studies = 1)** Dubuisson et al. (2012)							
Recent weight change	Questionnaire	↑[1]			0	22	**1**
Recent weight gain	Questionnaire			↔[1]	0	22	**1**
**Compliance (n studies = 1)** Dubuisson et al. (2012)							
Adherence to treatment	Investigation	↑[1]			0	22	**1**
Compliance to adjuvant therapies	Questionnaire	↑[1]			0	22	**1**
**Psychology (n studies=8)** Augustijn et al. (2018), Dubuisson et al. (2012), Eichen et al. (2018), Halberstadt et al. (2017), Mölbert et al. (2016), Pauli-Pott et al. (2010), Pott et al. (2010), Van Egmond-Froehlich et al. (2012)							
**Mental health (n studies = 4)** Dubuisson et al. (2012), Mölbert et al. (2016), Pott et al. (2010), Van Egmond-Froehlich et al. (2012)							
Conduct problem	Parent-rated Strength and Difficulty Questionnaire (SDQ)			↔[1]	0	22	**1**
Eating disorder	Pathology, Eating Questionnaire for Children: eating disinhibition			↔[2]	0	21	**2**
Mental health	Homemade Questionnaire, depression, depressive symptoms			↔[3]	0	21	**2**
Stress	Bad quality of sleep			↔[2]	0	21	**2**
**Impuls Control (n studies=5)** Augustijn et al. (2018), Eichen et al. (2018), Halberstadt et al. (2017), Pauli-Pott et al. (2010), Van Egmond-Froehlich et al. (2012)							
Digit Span	WISC-IV for children			↔[1]	0	22	**1**
Attention shifting and Inattention	Cambridge Neuropsychological Test Automated Battery: Intra-extra dimensional shift (IED): IED pre-ED errors, IED total trials (adjusted), Hyperactivity/Inattention: Parent rated SDQ; Go-NoGo procedure; incompatibility task		↓[2]	↔[3]	0	20	**3**
Self-regulation	Inhibitory control: Stop Signal Task: failures BL and after I; sensitivity to reward BL and after I			↔[5]	0	21	**2**
Impulsivity	Go/ No-Go procedure, incompatibility task	↑[1]			0	22	**1**
Updating abilities an inhibition control	Rapid visual information processing (RVP): total hits, total false alarms Stop Signal Task: failures after I, BL			↔[3]	0	20	**3**
Planning and decision making	Stockings of Cambridge (SOC): problem solved in minimum moves, mean moves, mean initial thinking time (ms), mean subsequent thinking time (ms); Wisconsin Card Sorting Test (WCST): Perseverative Errors			↔[5]	0	21	**2**
**Motivation (n studies=1)** Dubuisson et al. (2012)							
Child's motivation	Homemade Questionnaire	↑[1]			0	22	**1**
**Body Image (n studies=1)** Mölbert et al. (2016)							
Body size estimation				↔[1]	0	22	**1**
Tactile size estimation				↔[1]	0	22	**1**
Heart beat detection				↔[1]	0	22	**1**
Concerns about body weight and shape	Questionnaire			↔[1]	0	22	**1**

**Environmental (n studies=5)** Dubuisson et al. (2012), Eichen et al. (2018), Pott et al. (2009), Röbl et al. (2013), Van Egmond-Froehlich et al. (2012)							
**Treatment (n studies=2)** Dubuisson et al. (2012), Röbl et al. (2013)							
Number of visits	Documentation			↔[1]	22	0	**1**
Lengths of intervention	Documentation	↑[1]		↔[1]	4	17	**2**
**Social support (n studies=2)** Dubuisson et al. (2012), Pott et al. (2009)							
Social integration	Homemade Questionnaire			↔[1]	0	22	**1**
Family project-encouragement	Homemade Questionnaire	↑[1]			0	22	**1**
Family encouragement to leisure activities	Homemade Questionnaire			↔[1]	0	22	**1**
Maternal attachment attitude	Homemade Questionnaire			↔[1]	0	22	**1**
Parents' attendance	At home after school, dual parent household: Homemade Questionnaire			↔[2]	0	22	**1**
Breastfeeding (>= 6 months)	Homemade Questionnaire			↔[1]	0	22	**1**
**Family (n studies=4)** Dubuisson et al. (2012), Eichen et al. (2012), Pott et al. (2009), Van Egmond-Froehlich et al. (2012)							
Parents' executive functions	decision making: Wisconsin Card Sorting Test (WCST): perseverative errors, Stop Signal Task; Digit span: WAIS-IV			↔[3]	0	22	**1**
Maternal depression	Center for Epidemiologic Studies Depression Scale			↔[1]	0	22	**1**
Maternal BMI	Self-report			↔[1]	0	22	**1**
Only child	Homemade Questionnaire	↑[1]			0	22	**1**
Obesity in family	Homemade Questionnaire			↔[1]	0	22	**1**
Obese siblings	Questionnaire		↓[1]		0	22	**1**

**BL: Baseline, I: Intervention, N.R.: not reported, N.A.: not applicable, n: numbers of studies, numbers at the arrows map the amount of investigations*

Supplement 4: Overview Predictors BWLM

**Supplement 4: Overview Predictors Body Weight Loss Maintenance (BWLM)**							
Boutelle et al. (2019), Celi et al. (2003), Eichen et al. (2012), García-Calzón et al. (2014), Halberstadt et al. (2017), Murer et al. (2011), Van Egmond-Froehlich et al. (2012), Wolters et al. (2012)							
**Predictor**	**Measurement**	**Significant prediction of BWLM**			**N.R.**	**N.A.**	**n**
		positive ($\uparrow$)	negative ($\downarrow$)	none ($\leftrightarrow$)			
**Physiology (n studies = 4)** Celi et al. (2003), García-Calzón et al. (2014), Murer et al. (2011), Wolters et al. (2012)							
**Demographics (n studies = 1)** Celi et al. (2003)							
Sex	Interview/ Questionnaire			$\leftrightarrow^{2}$	7	0	**1**
Age	Interview/ Questionnaire			$\leftrightarrow^{1}$	7	0	**1**
Delayed puberty	Tanner stage		$\downarrow^{1}$		0	7	**1**
BMI z-core	measurement at BL and after I			$\leftrightarrow^{2}$	7	0	**1**
**Blood parameters (n studies = 3)** Celi et al. (2003), Murer et al. (2011), Wolters et al. (2012)							
Baseline leptin level	Blood measurements		$\downarrow^{1}_{6\ months}$	$\leftrightarrow^{2}_{12\ months}$	0	6	**2**
Leptin level after I	Blood measurements	$\uparrow^{1}$			0	7	**1**
Leptin decrease during I	Blood measurements	$\uparrow^{2}_{6,\ 12\ months}$		$\leftrightarrow^{2}_{2,\ 6\ or\ 12\ months}$	0	6	**2**
TSH decrease during I	Blood measurements			$\downarrow^{1}$	0	7	**1**
fT3 decrease during I	Blood measurements		$\downarrow^{1}$		0	7	**1**
fT4 decrease during I	Blood measurements			$\leftrightarrow^{1}$	0	7	**1**
Blood lipids	Blood measurements			$\leftrightarrow^{1}$	0	7	**1**
Blood pressure	Blood measurements			$\leftrightarrow^{1}$	0	7	**1**
Baseline Insulin level	Blood measurements			$\leftrightarrow^{1}$	0	7	**1**
**Genetics (n studies = 1)** García-Calzón et al. (2014)							
Telomere length baseline girls	Quantitative real-time polymerase chain reaction			$\leftrightarrow^{1}$	0	7	**1**
Telomere length baseline boys	Quantitative real-time polymerase chain reaction: mean and median split	$\uparrow^{2}$			0	7	**1**
**Behaviour (n studies = 2)** Celi et al. (2003), Boutelle et al. (2019)							
**Eating behaviour (n studies = 1)** Boutelle et al. (2019)							
Phenotype appetitive trajectories group: High Satiety	Grouping retrospective: Satiety vs. Emotional vs. Food, based on Questionnaires: Child eating Behaviour Questionnaire, Emotional Eating Scale for Children, Eating in the Absence of Hunger for Children	$\uparrow^{1}$			0	7	**1**
**Weight history (n studies = 1)** Celi et al. (2003)							
BMI z-score change	Weight change during I			$\leftrightarrow^{1}$	7	0	**1**
**Psychology (n studies = 3)** Eichen et al. (2018), Halberstadt et al. (2017), Van Egmond-Froehlich et al. (2012)							
**Mental health (n studies = 1)** Van Egmond-Froehlich et al. (2012)							
Conduct problem	Parent-rated Strength and Difficulty Questionnaire			$\leftrightarrow^{1}$	0	7	**1**
Eating disinhibition	Eating Questionnaire for Children: eating disinhibition			$\leftrightarrow^{1}$	0	7	**1**

**Impuls control (n studies = 3)** Eichen et al. (2018), Halberstadt et al. (2017), Van Egmond-Froehlich et al. (2012)							
Digit Span	WISC-IV for children			$\leftrightarrow^{2}_{6,\ 12\ months}$	0	7	**1**
Intention	Cambridge Neuropsychological Test Automated Battery: IED pre-ED errors, IED total trials (adjusted); Go-NoGo procedure; incompatibility task			$\leftrightarrow^{1}$	0	6	**2**
Self-regulation	Inhibitory control: Stop Signal Task: failures BL; sensitivity to reward			$\leftrightarrow^{4}_{6,\ 12\ months}$	0	6	**2**
Planning and decision making	Wisconsin Card Sorting Test (WCST): Perseverative Errors	$\uparrow^{1}_{12\ months}$		$\leftrightarrow^{1}_{6\ months}$	0	7	**1**
**Environment (n studies = 3)** Celi et al. (2003), Eichen et al. (2012), Van Egmond-Froehlich et al. (2012)							
**Treatment (n studies = 8)** Boutelle et al. (2019), Celi et al. (2003), Eichen et al. (2012), García-Calzón et al. (2014), Halberstadt et al. (2017), Murer et al. (2011), Van Egmond-Froehlich et al. (2012), Wolters et al. (2012)							
Lengths of intervention	Documentation	N.R.	N.R.	N.R.	9	0	**0**
**Family (n studies = 3)** Celi et al. (2003), Eichen et al. (2012), Van Egmond-Froehlich et al. (2012)							
Parents' cognition	Digit span: WAIS-IV			$\leftrightarrow^{2}_{6,\ 12\ months}$	0	7	**1**
Parents' executive functions	Wisconsin Card Sorting Test (WCST), Stop Signal Task			$\leftrightarrow^{2}_{6,\ 12\ months}$	0	7	**1**
Parental BMI	Self-report			$\leftrightarrow^{2}$	0	6	**2**

**BL: Baseline, I: Intervention, N.R.: not reported, N.A.: not applicable, n: numbers of studies, numbers at the arrows map the amount of investigations, IED*: Intra-extra dimensional shift

Supporting Text S1: Search Strategy

(PubMed [p. 1-2], Cochrane Library [p. 3-6], Web of science [p.7-8])

<u>Term PubMed</u>

(((("Adiposity"[Mesh] OR Adipos*[tiab] OR Obesity[tiab] OR Adipose[tiab] OR Obese[tiab] OR Overweight [tiab] OR "Obesity"[Mesh] OR "pediatric obesity"[MeSH])) AND (("Weight Reduction Programs"[Mesh] OR "Diet, Reducing" [Mesh] OR Slimming program [tiab] OR Weight-loss program [tiab] OR Weight-loss intervention[tiab] OR Weight loss program[tiab] OR Weight loss therapy[tiab] OR Weight loss management[tiab] OR Weight reduction program[tiab] OR Weight reduction therapy[tiab] OR Weight reduction management[tiab] OR Weight reduction intervention[tiab] OR Weight reducing program[tiab] OR Weight reducing therapy[tiab] OR Weight reducing management[tiab] OR Weight reducing intervention[tiab] OR Weight control program[tiab] OR Weight control therapy[tiab] OR Weight control management[tiab] OR Weight control intervention[tiab]))) AND (((("Weight Loss"[Mesh:NoExp] OR Weight Loss*[tiab] OR Weight Reduction*[tiab] OR "Reductions of Weight"[tiab] OR "Reduction of Weight"[tiab] OR "Loss of Weight"[tiab] OR "Losses of Weight"[tiab]) AND (Variable*[tiab] OR Factor[tiab] OR Factors[tiab] OR Prognos*[tiab] OR Mediator*[tiab] OR follow-up[tiab]OR predict*[tiab] OR relationship[tiab] OR Indicat*[tiab] OR Correl*[tiab] OR Mediate*[tiab] OR Associat*[tiab] OR Influence[tiab] OR Impact[tiab] OR Effect[tiab] OR Effects [tiab] OR Role[tiab])) OR Weight Predictors[tiab] OR "Weight Predictor"[tiab] OR "Weight trajectory predictors"[tiab] OR "Weight trajectory predictor"[tiab] OR Weight outcome*[tiab] OR (("Gastrointestinal Tract"[Mesh] OR Gastrointestinal Tracts[tiab] OR Gastrointestinal Tract[tiab] OR Digestive Tract[tiab] OR Digestive Tracts[tiab]) AND ("Smell"[Mesh] OR Smell[tiab] OR Olfaction[tiab] OR Olfactory[tiab] OR "Olfaction Disorders"[Mesh] OR Cacosmia[tiab] OR Cacosmias[tiab] OR Dysosmia[tiab] OR Dysosmias[tiab] OR Paraosmia[tiab] OR Paraosmias[tiab] OR Anosmia[tiab] OR "Taste"[Mesh] OR Taste[tiab] OR Tastes[tiab] OR Gustation[tiab] OR Gustations[tiab] OR "Taste Disorders"[Mesh] OR ("Feeding Behavior"[Mesh] NOT (Cannibalism[Mesh] OR Coprophagia[Mesh])) OR Eating Behavior[tiab] OR Eating Behaviors[tiab] OR Food Habits[tiab] OR Food Habit[tiab] OR Eating Habits[tiab] OR Eating Habit[tiab] OR Dietary Habits[tiab] OR Dietary Habit[tiab] OR Diet Habits[tiab] OR Diet Habit[tiab])) OR (("Autonomic Nervous System"[Mesh] OR Autonomic Nervous Systems[tiab] OR Autonomic Nervous System[tiab]) AND (Heart Rate[tiab] OR Heart Rates[tiab] OR "Heart beat perception"[tiab] OR ("Hemodynamics"[Mesh] NOT (Baroreflex[Mesh] OR Pulmonary Wedge Pressure[Mesh] OR Valsalva Maneuver[Mesh] OR Ventricular Pressure[Mesh])))) OR "Depression"[Mesh] OR Depressions[tiab] OR Depresson[tiab] OR Depressive Symptoms[tiab] OR Depressive Symptom[tiab] OR "Depressive Disorder"[Mesh] OR Depressive Disorders[tiab] OR Depressive Disorder[tiab] OR Depressive Neuroses[tiab] OR Depressive Neurosis[tiab] OR Endogenous Depression[tiab] OR Endogenous Depressions[tiab] OR Depressive Syndrome[tiab] OR Depressive Syndromes[tiab] OR "Anxiety"[Mesh:NoExp] OR Hypervigilance[tiab] OR Nervousness[tiab] OR Anxiety[tiab] OR Anxieties[tiab] OR "Anxiety Disorders"[Mesh] OR "Self Concept"[Mesh] OR Self Perception[tiab] OR Self Perceptions[tiab] OR Self Confidence[tiab]OR Self Esteem[tiab] OR Self Esteems[tiab] OR "Body Image"[Mesh] OR Body Images[tiab] OR Body Image[tiab] OR Body Representation[tiab] OR Body Representations[tiab] OR Body Schema[tiab] OR Body Schemas[tiab] OR "Stress, Psychological"[Mesh] OR Stress[tiab] OR Stresses[tiab] OR Suffering[tiab] OR Sufferings[tiab] OR "Physical Fitness"[Mesh] OR "Physical Endurance"[Mesh] OR Physical Stamina[tiab] OR Physical Endurance[tiab] OR Physical Fitness[tiab] OR "metabolism" [Subheading] OR "Genetics"[Mesh]))) AND (((("Adolescent"[Mesh] OR "Child"[Mesh] OR "Infant"[Mesh] OR Infan* [tiab] OR minors[tiab] OR minors*[tiab] OR boy[tiab]

OR boys[tiab] OR boyhood[tiab] OR girl*[tiab] OR kid[tiab] OR kids[tiab] OR child*[tiab] OR adolescen*[tiab] OR juvenile*[tiab] OR youth*[tiab] OR teen*[tiab] OR tween*[tiab] OR underage*[tiab] OR prepubescen*[tiab] OR prebubescen*[tiab] OR pediatrics[tiab] OR pediatric*[tiab] OR peadiatric*[tiab] OR young people*[tiab] OR "young person"[tiab] OR Childhood[tiab]) NOT ("Adult"[Mesh] NOT ("Adolescent"[Mesh] OR "Child"[Mesh] OR "Infant"[Mesh] OR Infan* [tiab] OR minors[tiab] OR minors*[tiab] OR boy[tiab] OR boys[tiab] OR boyhood[tiab] OR girl*[tiab] OR kid[tiab] OR kids[tiab] OR child*[tiab] OR adolescen*[tiab] OR juvenile*[tiab] OR youth*[tiab] OR teen*[tiab] OR tween*[tiab] OR underage*[tiab] OR prepubescen*[tiab] OR prebubescen*[tiab] OR pediatrics[tiab] OR pediatric*[tiab] OR peadiatric*[tiab] OR young people*[tiab] OR "young person"[tiab] OR Childhood[tiab]))) NOT ("Animals"[Mesh] NOT "Humans"[Mesh])))

Term Cochrane Library

#1

[mh "Adiposity"] OR<br>(Adipos* OR<br>Obesit* OR<br>Obes* OR<br>Overweight OR):ti,ab,kw<br>[mh "Obesity"] OR<br>[mh "pediatric obesity"]

#2

[mh "Weight Reduction Programs"] OR<br>[mh "Diet, Reducing"] OR<br>("Slimming program" OR<br>"Weight-loss program*" OR<br>"Weight-loss intervention*" OR<br>"Weight loss program*" OR<br>"Weight loss therapy" OR<br>"Weight loss management" OR<br>"Weight reduction program*" OR<br>"Weight reduction therapy*" OR<br>"Weight reduction management" OR<br>"Weight reduction intervention*" OR<br>"Weight reducing program*" OR<br>"Weight reducing therapy" OR<br>"Weight reducing management" OR<br>"Weight reducing intervention*" OR<br>"Weight control program*" OR<br>"Weight control therapy" OR<br>"Weight control management" OR<br>"Weight control intervention*"):ti,ab,kw

#3

(([mh "Weight Loss"] OR<br>("Weight Loss*" OR<br>"Weight Reduction*" OR<br>"Reductions of Weight" OR<br>"Reduction of Weight" OR<br>"Loss of Weight" OR<br>"Losses of Weight"):ti,ab,kw) AND<br>((Variable* OR<br>Factor OR<br>Factors OR<br>Prognos* OR<br>Mediator* OR<br>follow-up OR<br>predict* OR<br>relationship OR<br>Indicat* OR<br>Correl* OR<br>Mediate* OR<br>Associat* OR

Influence OR<br>Impact OR<br>Effect OR<br>Effects OR<br>Role):ti,ab,kw)) OR<br>("Weight Predictor*" OR<br>"Weight trajectory predictor*" OR<br>"Weight outcome*"):ti,ab,kw

#4

(([mh "Gastrointestinal Tract"] OR<br>("Gastrointestinal Tract*" OR<br>"Digestive Tract*"):ti,ab,kw) AND<br>([mh "Smell"] OR<br>[mh "Olfaction Disorders"] OR<br>[mh "Taste"] OR<br>[mh "Taste Disorders"] OR<br>(Smell OR<br>Olfaction OR<br>Olfactory OR<br>Cacosmia* OR<br>Dysosmia* OR<br>Paraosmia* OR<br>Anosmia OR<br>Taste* OR<br>Gustation* OR<br>"Eating Behavior*" OR<br>"Food Habit*" OR<br>"Eating Habit*" OR<br>"Dietary Habit*" OR<br>"Diet Habit*"):ti,ab,kw OR<br>([mh "Feeding Behavior"] NOT ([mh "Cannibalism"] OR [mh "Coprophagia"])))) OR<br>(([mh "Autonomic Nervous System"] OR<br>("Autonomic Nervous System*"):ti,ab,kw)<br>AND<br>(("Heart Rate*" OR<br>"Heart beat perception"):ti,ab,kw OR<br>([mh "Hemodynamics"] NOT ([mh "Baroreflex"] OR [mh "Pulmonary Wedge Pressure"] OR [mh "Valsalva Maneuver"] OR [mh "Ventricular Pressure"])))) OR<br>[mh "Depression"] OR<br>[mh "Depressive Disorder"] OR<br>[mh "Anxiety"] OR<br>[mh "Anxiety Disorders"] OR<br>[mh "Self Concept"] OR<br>[mh "Body Image"] OR<br>[mh "Stress, Psychological"] OR<br>[mh "Physical Fitness"] OR<br>[mh "Physical Endurance"] OR<br>[mh "Genetics"] OR<br>(Depressions* OR

"Depressive Symptom*" OR
"Depressive Disorder*" OR
"Depressive Neuroses" OR
"Depressive Neurosis" OR
"Endogenous Depression*" OR
"Depressive Syndrome*" OR
Hypervigilance OR
Nervousness OR
Anxiety OR
Anxieties OR
"Self Perception*" OR
"Self Confidence" OR
"Self Esteem*" OR
"Body Image*" OR
"Body Representation*" OR
"Body Schema*" OR
Stress* OR
Suffering* OR
"Physical Stamina" OR
"Physical Endurance" OR
"Physical Fitness"):ti,ab,kw

#5

([mh "Adolescent"]
OR [mh "Child"]
OR [mh "Infant"]
OR (Infan*
OR minors*
OR boy*
OR boyhood
OR girl*
OR kid*
OR child*
OR adolescen*
OR juvenile*
OR youth*
OR teen*
OR tween*
OR underage*
OR prepubescen*
OR prebubescen*
OR pediatric*
OR peadiatric*
OR "young people*"
OR "young person"
OR Childhood):ti,ab,kw) NOT
([mh "Adult"] NOT ([mh "Adolescent"]
OR [mh "Child"]
OR [mh "Infant"]
OR (Infan*
OR minors*
OR boy*
OR boyhood
OR girl*

OR kid*
OR child*
OR adolescen*
OR juvenile*
OR youth*
OR teen*
OR tween*
OR underage*
OR prepubescen*
OR prebubescen*
OR pediatric*
OR peadiatric*
OR "young people*"
OR "young person"
OR Childhood):ti,ab,kw)) NOT
([mh "Animals"] NOT
[mh "Humans"])

#6

#3 OR #4

STRATEGY:

#1 AND #2 AND #5 AND #6

Term Web of Science

#1

TS=(Adipos* OR
Obesit* OR
Obese OR
Overweight)

#2

TS=(Weight NEAR/0 (Reduction OR Loss OR Reducing OR Control*) NEAR/0 (Program$ OR Management OR Intervention$ OR Therap*))

#3

TS=(
(((Weight NEAR/0 (Loss OR Reduction$)) OR ((Reduction$ OR Loss) NEAR/0 Weight)) AND
(Variable* OR Factor$ OR Prognos* OR Mediator$ OR follow-up OR predict* OR relationship OR Indicat* OR Correl* OR Mediate* OR Associat* OR Influence OR Impact$ OR Effect$ OR Role$)) OR
(((Weight NEAR/0 (Predictor$ OR outcome$)) OR (Weight NEAR/0 Trajectory NEAR/0 Predictor$))))

#4

TS=(
(((Gastrointestinal OR Digestive) NEAR/0 Tract$) AND
(Smell OR Olfactor* OR Cacosmia$ OR Dysomia$ OR Parasomia OR Anosmia$ OR Taste$ OR Gustation$ OR (Eating NEAR/0 Behavior$) OR ((Food OR Dietary OR Eating) NEAR/0 Habit$))) OR

((Autonomic NEAR/0 Nervous NEAR/0 System$) AND
((Heart NEAR/0 Rate$) OR (Heart NEAR/0 Beat Near/0 Perception$) OR Hemodynamic*)) OR

(Depression$ OR (Depressive NEAR/0 (Disorder$ OR Symptom$ OR Neuros?s OR Syndrome$)) OR (Endogenous NEAR/0 Depression$) OR Anxiet* OR Hypervigiliance$ OR Nervousness OR (Self NEAR/0 (Concept OR Perception$ OR Confidence OR Esteem$)) OR (Body NEAR/0 (Image$ OR Representation$ OR Schema$)) OR stress OR stresses OR Suffering$ OR (Physical NEAR/0 (Fitness OR Endurance OR Stamina))))

#5

TS=(
((Adolescen* OR Child OR Children OR Minor$ OR Infant$ OR Boy$ OR Girl$ OR Boyhood OR Kid$ OR Juvenile$ OR Youth* OR Teen$ OR Underage$ OR Pre?ubescen* OR Pediatric* OR Peadiatric* OR (Young NEAR/0 (Person$ OR People)) OR Childhood) NOT
(Adult$ NOT (Adolescen* OR Child OR Children OR Minor$ OR Infant$ OR Boy$ OR Girl$ OR Boyhood OR Kid$ OR Juvenile$ OR Youth* OR Teen$ OR Underage$ OR Pre?ubescen* OR Pediatric* OR Peadiatric* OR (Young NEAR/0 (Person$ OR People)) OR Childhood))) NOT
(Animal$ NOT Human$))

#6

#3 OR #4

STRATEGY:

#1 AND #2 AND #5 AND #6
